# A TaSnRK1α Modulates TaPAP6L‐Mediated Wheat Cold Tolerance through Regulating Endogenous Jasmonic Acid

**DOI:** 10.1002/advs.202303478

**Published:** 2023-09-22

**Authors:** Lingran Zhang, Ning Zhang, Sisheng Wang, Hongyan Tian, Lu Liu, Dan Pei, Xiaodong Yu, Lei Zhao, Feng Chen

**Affiliations:** ^1^ National Key Laboratory of Wheat and Maize Crop Science / CIMMYT‐China Wheat and Maize Joint Research Center /Agronomy College Henan Agricultural University Zhengzhou 450046 China

**Keywords:** cold stress, common wheat, jasmonic acid, phosphorylation, TaPAP6L, TaSnRK1α

## Abstract

Here, a sucrose non‐fermenting‐1‐related protein kinase alpha subunit (TaSnRK1α‐1A) is identified as associated with cold stress through integration of genome‐wide association study, bulked segregant RNA sequencing, and virus‐induced gene silencing. It is confirmed that TaSnRK1α positively regulates cold tolerance by transgenes and ethyl methanesulfonate (EMS) mutants. A plastid‐lipid‐associated protein 6, chloroplastic‐like (TaPAP6L‐2B) strongly interacting with TaSnRK1α‐1A is screened. Molecular chaperone DJ‐1 family protein (TaDJ‐1‐7B) possibly bridged the interaction of TaSnRK1α‐1A and TaPAP6L‐2B. It is further revealed that TaSnRK1α‐1A phosphorylated TaPAP6L‐2B. Subsequently, a superior haplotype TaPAP6L‐2B^30S^ ^/38S^ is identified and confirmed that both R30S and G38S are important phosphorylation sites that influence TaPAP6L‐2B in cold tolerance. Overexpression (OE) and EMS‐mutant lines verified TaPAP6L positively modulating cold tolerance. Furthermore, transcriptome sequencing revealed that *TaPAP6L‐2B‐OE* lines significantly increased jasmonic acid (JA) content, possibly by improving precursor α‐linolenic acid contributing to JA synthesis and by repressing JAR1 degrading JA. Exogenous JA significantly improved the cold tolerance of wheat plants. In summary, TaSnRK1α profoundly regulated cold stress, possibly through phosphorylating TaPAP6L to increase endogenous JA content of wheat plants.

## Graphical abstract

Under the exposure to cold stress for wheat plants, TaSnRK1α enriching to the chloroplast with the help of TaDJ‐1 phosphorylated TaPAP6L‐2B^R30S/G38S^ and thereby caused the accumulation of JA (jasmonic acid) content. Finally, wheat plants with TaPAP6L^R30S/G38S^ showed cold tolerance due to the phosphorylation by TaSnRK1α, whereas wheat plants with TaPAP6L^R30/G38^ showed cold sensitivity.

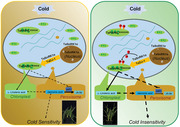

## 1 Introduction

Wheat is one of the most widely cultivated major food crops. However, cold stress is one of the key abiotic stresses that damage wheat growth and cause severe yield loss in common wheat.^[^ ^1^, ^2^ ^]^ Numerous studies have demonstrated that the probability and potential risk of extreme cold events are gradually increasing as a result of global warming. The discovery of genes modulating cold stress for the development of cold‐tolerant wheat varieties is an effective method to prevent cold injury in wheat.

Previous studies have identified some important cold stress genes in plants, including transcription factors (*CBF*, *MYB*, *ZIPI*, and *DREB*), protein kinases (*MPK, OST1*), and cold response protein chilling‐tolerance divergence 1 (*COLD1*), etc. The CBF/DREB‐mediated transcriptional regulatory cascade has been shown to be the major cold signaling pathway, and this cascade is essential to activate a set of cold responsive genes.^[^ ^3^, ^4^ ^]^ The *MPK3/6‐ICE1* and *ICE‐CBF‐COR* models are also widely reported to regulate plant hypothermia response.^[^ ^5^, ^6^ ^]^ MPK3/MPK6 phosphorylates and destabilizes the inducer of CBF expression 1(ICE1), thereby negatively regulates CBF expression and frost resistance in plants.^[^ ^7^, ^8^, ^9^ ^]^ Plant U‐box E3 ligases PUB25 and PUB26 dynamically modulate ICE1 stability via differential ubiquitination during cold stress in Arabidopsis.^[^ ^10^ ^]^ *COLD1* interacts with the G‐protein *α* subunit to activate the Ca^2+^ channel for sensing low temperature and to accelerate G‐protein GTPase activity.^[^ ^11^ ^]^ The Ca^2+^ transporter ANNEXIN1 was found to mediate cold‐induced calcium signaling and freezing tolerance in plants.^[^ ^12^ ^]^ A recent study indicated that loss‐of‐function mutations of *COLD1* caused reduced rice chilling tolerance, and natural variation of codon repeats in *COLD11* endows rice with chilling resilience.^[^ ^13^ ^]^ Overexpression of glucose‐6‐phosphate dehydrogenase, 6‐phosphogluconate dehydrogenase, and trehalose 6‐phosphate synthase 11 from winter wheat enhanced the cold tolerance in Arabidopsis, showing a potential value of these genes in wheat cold‐tolerance breeding.^[^ ^14^, ^15^, ^16^ ^]^ The plastid‐lipid‐associated protein 6 (*PAP6*)‐silenced wheat plants showed significantly decreased cold tolerance.^[^ ^17^ ^]^ Silencing of glycine‐rich RNA‐binding protein 2, CBS domain‐containing protein chloroplastic‐like, and cold acclimation protein Wcor410c reduced cold tolerance in common wheat.^[^ ^18^ ^]^ Overexpression and silencing of the gene encoding this DHN 13 in Arabidopsis and wheat led to increased tolerance and sensitivity to cold stress, respectively.^[^ ^19^ ^]^

Phytohormones are important regulators of plant growth and development, as well as abiotic stress response signal networks, suggesting that plant hormones interact with signals for plant growth and environmental stresses.^[^ ^14^ ^]^ Phytohormone regulation in wheat cold stress is another strategy to study wheat cold tolerance. Exogenous abscisic acid (ABA) enhances cold tolerance, possibly by increasing the activity of antioxidant enzymes.^[^ ^20^, ^21^ ^]^ Initiation of melatonin during grain filling of mother plants may improve the cold tolerance of wheat offspring at the seedling stage.^[^ ^22^ ^]^ During the recovery period of wheat after cold stress, foliar spraying of melatonin enhances the cold tolerance of wheat plants.^[^ ^23^ ^]^ Jasmonic acid (JA) regulates organ development by affecting the synthesis of other secondary metabolites and also regulates plant responses to drought, cold, ozone, and ultraviolet stress.^[^ ^24^, ^25^, ^26^, ^27^ ^]^ JA positively regulates cold tolerance by promoting ABA biosynthesis in tomato.^[^ ^28^ ^]^ Fibrillins (FBNs), generally described as plastid lipid‐associated proteins (PAPs), are the most abundant plastidial proteins.^[^ ^29^ ^]^ Some evidences suggest that FBNs are involved in phytohormone signaling, especially JA signaling.^[^ ^30^, ^31^ ^]^ Arabidopsis fibrins *FBN1a, FBN1b*, and *FBN2* regulate JA synthesis induced by light or cold stress.^[^ ^32^ ^]^ Many studies have shown that FBN family proteins may participate in the regulation of JA pathway in some way.^[^ ^33^, ^34^, ^35^ ^]^ Plants with JA pretreatment increased significantly the activity of antioxidant enzymes, and thereby improved the cold tolerance.^[^ ^36^, ^37^ ^]^ JA was also reported to positively regulate downstream cold response genes in the CBF transcription pathway and ultimately enhanced cold tolerance.^[^ ^38^ ^]^

In this study, we found that the expression of sucrose non‐fermenting‐1‐related protein kinase alpha subunit (*TaSnRK1α*) alleviates the damage of cold stress in wheat plants. We further found that TaSnRK1α interacts with PAP6, chloroplastic‐like (TaPAP6L) and promotes its accumulation by phosphorylation. Moreover, our results indicate that *TaSnRK1α* and *TaPAP6L* increase the content of plant hormone JA, thereby regulating plants to prevent the damage from cold stress. Consequently, our study reveals the important role of *TaSnRK1α* in regulating wheat resistance to cold stress.

## 2 Results

### 2.1 Identification of Candidate Genes Associated with Wheat Cold Stress by GWAS

**Figure 1:**
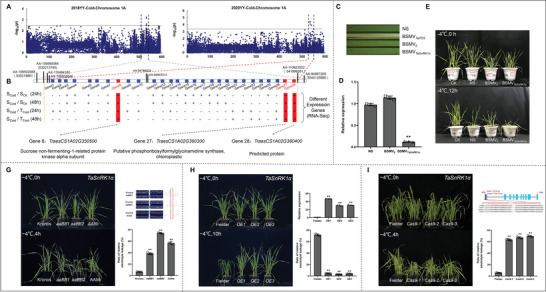
*TaSnRK1α−1A* was identified by GWAS, BSR‐seq and VIGS; *TaSnRK1α−1A* positively regulated wheat cold tolerance. A) GWAS results revealed an important genetic locus gCTI‐1A (532‐541 Mb) regulating cold tolerance on chromosome 1A in two environments (2018 and 2020 in Yuanyang). B) Expressions of 28 annotation genes in a 9‐Mb (532‐541 Mb) interval between cold‐tolerant pools (CTPs) and cold‐sensitive pools (CSPs) based on transcriptome analysis. “+” indicated differentially expressed genes (DEGs); “‐” indicated none DEG. S_Cold_: cold‐sensitive pools under cold stress for 24 h or 48 h. S_CK_: cold‐sensitive pools without cold stress. T_Cold_: cold‐tolerant pools under cold stress for 24 h or 48 h. C–E) VIGS (virus‐induced gene silencing) experiment verified the function of *TaSnRK1α−1A* gene. C,D) Phenotypes of wild type (WT), BSMV_0_, and BSMV*_TaSnRK1α_* plants (*n* = 5 plants per replicate) before and after cold stress; E) the relative expression levels (*n* = 3). CK represents wheat plants without cold stress; NS represents unsilenced plants. G) Mutation sites of *TaSnRK1α* in Kronos EMS mutants and a comparison of phenotype and physiological indices of mutants and the controls (*n* = 5) under cold stress. H) The relative expression of *TaSnRK1α−1A* in *TaSnRK1α−1A‐OE* lines (*n* = 3) and a comparison of phenotype and physiological indices of *TaSnRK1α−1A‐OE* lines and the controls (*n* = 5) under cold stress. I) The editing sites in *TaSnRK1α*‐edited lines and a comparison of phenotype and physiological indices of *TaSnRK1α*‐edited lines and the controls (*n* = 5) under cold stress. Values are presented as mean ± SE. Statistical significance was determined by a two‐sided *t*‐test (* *P* < 0.05, ** *P* < 0.01).

Genome‐wide association analysis (GWAS) was conducted for cold tolerance index (CTI) in an association panel consisting of 243 wheat accessions previously genotyped using Wheat 660K SNP array.^[^ ^39^ ^]^ GWAS identified an important genetic locus *gCTI‐1A* significantly associated with cold stress on chromosome 1A. To further map *gCTI‐1A*, we re‐run GWAS using only SNPs on 1A genome, and found that the *gCTI‐1A* containing five significant SNPs was in a 9‐Mb (532‐541 Mb) interval containing 28 annotation genes (**Figure** **1**A). BSR‐Seq (bulked segregant RNA sequencing) analysis in a Recombinant Inbred Lines (RIL) (Shanghai3/Catbird×Naxos, SC) population indicated that 13 of the 28 genes were significantly induced at both 24 h and 48 h after cold stress, and 3 (*TraesCS1A02G350500*, *TraesCS1A02G360300*, and *TraesCS1A02G360400*) of these 13 genes showed differential expression between cold‐tolerant and cold‐sensitive pools at both 24 h and 48 h after cold treatment (Figure 1B; Table S1, Supporting Information).

### 2.2 VIGS Revealed the Role of TaSnRK1α Regulating Wheat Cold Stress

To confirm the *gCTI‐1A* gene, we silenced three above‐mentioned candidate genes in wheat plants by virus‐induced gene silencing (VIGS) using a Barley Stripe Mosaic Virus (BSMV) vector. After inoculation for 14 days, the relative expression levels were identified by qRT‐PCR, and the silenced‐plants were subjected to cold stress at −4 °C for 12 h. Results indicated that only *TraesCS1A02G350500*‐silenced plants among three candidates showed obvious drooping and wilting (Figure 1C–E; Figure S1, Supporting Information). The *TraesCS1A02G350500* encodes a sucrose non‐fermenting‐1‐related protein kinase alpha subunit, hereafter designated as *TaSnRK1α−1A* on 1A, was selected as candidate for further study.

### 2.3 Function Confirmation of TaSnRK1α by EMS Mutants, Overexpression, and CRISPR/Cas9

To further functional verification of *TaSnRK1α*, we screened Ethyl methanesulfonate (EMS)‐mutagenized tetraploid wheat Kronos library, and identified three mutants aaBB1 (K331), aaBB2 (K4218), and AAbb (K4220) of *TaSnRK1α*. We backcrossed these three mutants twice with wild type to obtain the BC_2_ lines, respectively. After cold stress (−4 °C, 4 h) for BC_2_ lines, all three mutants showed relatively serious drooping and dehydration, and possessed significantly increased relative electrolyte leakage rates compared with wild type (Figure 1G).

To determine whether the expression of *TaSnRK1α* contributes to cold tolerance in hexaploid wheat, we generated *TaSnRK1α−1A*‐overexpression (*TaSnRK1α−1A‐OE*) lines by transferring full‐length cDNA of *TaSnRK1α−1A* from cold tolerance parent Shanghai3/Catbird into hexaploid wheat cultivar Fielder. Three positively high‐expression lines detected by qRT‐PCR were self‐crossed into T_3_ generation and three T_3_ lines with high expression levels were selected for cold stress treatment. After cold stress (−4 °C, 10 h), *TaSnRK1α−1A*‐*OE* lines showed significantly stronger resistance to drooping and dehydration and possessed significantly decreased relative electrolyte leakage rate compared with wild type (Figure 1H).

To validate the effect of *TaSnRK1α* mutants in hexaploid wheat, we used CRISPR/Cas9‐mediated gene editing technique to knock out *TaSnRK1α* gene in Fielder. Mutation sites were confirmed through sequencing by Hi‐TOM and were further verified through Sanger sequencing (Table S2, Supporting Information).^[^ ^16^ ^]^ After cold stress (−4 °C, 6 h), *TaSnRK1α*‐edited wheat mutant lines showed more serious drooping and dehydration and possessed significantly increased relative electrolyte leakage rate compared with non‐edited wild type (Figure 1I). These results suggested that *TaSnRK1α* positively modulated wheat cold tolerance.

### 2.4 TaSnRK1α Interacted with TaPAP6L

**Figure 2:**
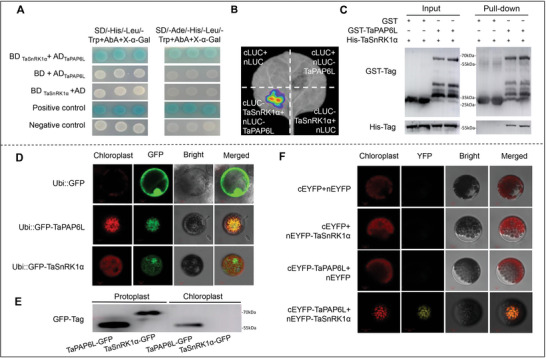
TaSnRK1α strongly interacted with TaPAP6L. A) Yeast two‐hybrid (Y2‐H) assays verified the interaction of TaSnRK1α−1A and TaPAP6L‐2B. AD‐T+BD‐53 group was used as positive control, while AD‐T+BD‐Lam group was used as negative controls. B) Firefly luciferase complementation imaging assay of the interaction between TaSnRK1α−1A and TaPAP6L‐2B in tobacco leaves. The empty vector groups were as negative controls. C) Pull‐down assay confirmed the interaction between GST‐TaSnRK1α and His‐TaPAP6L. D) Subcellular localization of the GFP‐TaSnRK1α and GFP‐TaPAP6L fusion protein in wheat protoplast. GFP fusion protein acts as the controls. E) Western blotting detected transient expression of Ubi::GFP‐TaSnRK1α or Ubi::GFP‐TaPAP6L in wheat protoplasts and chloroplasts, respectively. F) Bimolecular fluorescence complementary (BiFC) confirm the interaction between TaSnRK1α and TaPAP6L. cEYFP+nEYFP, cEYFP+nEYFP‐TaSnRK1α, cEYFP‐TaPAP6L+nEYFP were used as controls.

To dissect the regulatory mechanism of *TaSnRK1α* in cold tolerance, we conducted a yeast two‐hybrid (Y2‐H) to screen *TaSnRK1α−1A*‐interacting proteins using *TaSnRK1α−1A* as a bait in a wheat cDNA library. Among several interacting proteins, a client TaPAP6L‐2B (TraesCS2B02G171300, plastid‐lipid‐associated protein 6, chloroplastic‐like) on 2B was selected for further analysis because TaPAP6L is a member of the PAP/ FBNs protein family and it has been reported that this family was involved in plant response to temperature.^[^ ^17^, ^40^ ^]^ We next confirmed the interaction of TaSnRK1α−1A and TaPAP6L‐2B proteins in the yeast cells by Y2‐H (**Figure** **2**A) and in the tobacco leaves by Luciferase Reporter systems, respectively (Figure 2B). We then performed an in vitro pull‐down, and results indicated that GST‐TaPAP6L specifically pulled down TaSnRK1α‐His (Figure 2C). Together, these results indicated that TaSnRK1α−1A interacted with TaPAP6L‐2B in vivo and in vitro.

### 2.5 TaDJ‐1 as a Molecular Chaperone Possibly Bridged the Interaction of TaSnRK1α and TaPAP6L

To identify locations of TaSnRK1α−1A and TaPAP6L‐2B in cells, we performed subcellular localization in wheat protoplasts by transiently transforming them with Ubi::GFP‐TaSnRK1α or Ubi::GFP‐TaPAP6L, respectively. Results showed that TaSnRK1α−1A was mainly localized in the nucleus and cytoplasm, but TaPAP6L‐2B was in chloroplast (Figure 2D). We subsequently extracted wheat protoplast protein and chloroplast protein and imprinted them with anti‐GFP antibody, respectively. Results showed that both TaSnRK1α−1A and TaPAP6L‐2B could be detected in wheat protoplast protein, but only TaPAP6L‐2B could be detected in chloroplast protein, implying that TaSnRK1α−1A was not expressed in chloroplasts (Figure 2E). Bimolecular Fluorescent Complementary (BiFC) in wheat mesophyll protoplasts showed that TaSnRK1α−1A interacted with TaPAP6L‐2B on the chloroplast (Figure 2F).

**Figure 3:**
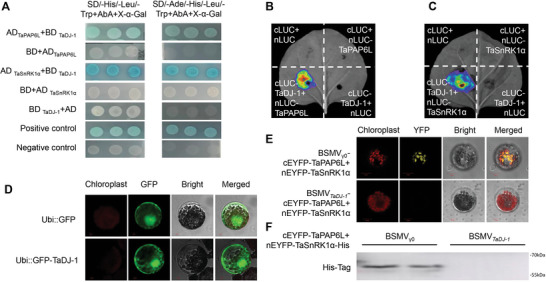
TaDJ‐1 as a molecular chaperone is involved in the interaction of TaSnRK1α and TaPAP6L. A) Yeast two‐hybrid (Y2‐H) assays to verify the interaction of TaDJ‐1‐7B with TaSnRK1α−1A and TaPAP6L‐2B. AD‐T+BD‐53 group was used as positive control, while AD‐T+BD‐Lam group was used as negative control. B) Firefly luciferase complementation imaging assay of the interaction between TaDJ‐1‐7B and TaPAP6L‐2B in tobacco leaves. The empty vector groups were as negative controls. C) Firefly luciferase complementation imaging assay of the interaction between TaDJ‐1‐7B and TaSnRK1α−1A in tobacco leaves. The empty vector groups were considered as negative controls. D) Subcellular localization of the TaDJ‐1‐7B protein in wheat protoplast. GFP fusion proteins were as controls. E) Bimolecular fluorescence complementation (BiFC) analysis of the interaction between TaSnRK1α‐His and TaPAP6L‐2B in the protoplasts of BSMV*_TaDJ‐1_* and BSMV_γ0_ plants. F) The expression of TaSnRK1α‐His protein in chloroplast proteins from protoplasts of BSMV_γ0_ and BSMV*_TaDJ‐1_* plants, respectively.

To address why TaSnRK1α−1A could move to the chloroplast and interact with TaPAP6L‐2B, we re‐analyzed the results of above‐mentioned screened proteins by TaSnRK1α−1A and identified a client molecular chaperone, TaDJ‐1‐7B (TraesCS7B02G345700, DJ‐1 family protein) on 7B. As a molecular chaperone, DJ‐1 was encoded by parkinson's disease 7 in human beings and has been reported to play an important role in chloroplast development and biotic and abiotic stresses response in plants.^[^ ^41^, ^42^, ^43^, ^44^, ^45^ ^]^ We subsequently verified the interaction of TaSnRK1α−1A and TaPAP6L‐2B with TaDJ‐1‐7B in the yeast cell by Y2‐H (**Figure** **3**A) and Luciferase Reporter systems, respectively (Figure 3B,C). Subcellular localization in wheat protoplasts showed that Ubi::GFP‐TaDJ‐1 was obviously observed in almost the whole cell (Figure 3D). Therefore, we assumed that TaDJ‐1, as a molecular chaperone, accompanied with TaSnRK1α−1A to the chloroplast by interaction. To verify this hypothesis, we silenced *TaDJ‐1* in wheat plants by VIGS and performed BiFC assay for verifying whether the interaction of TaSnRK1α‐His with TaPAP6L‐2B occurs using protoplasts from BSMV*_TaDJ‐1_* and BSMV_γ0_ plants. Results showed that YFP florescence signal was significantly weaker in BSMV*_TaDJ‐1_* plants than in BSMV_γ0_ plants (Figure 3E). Moreover, we extracted chloroplast proteins from protoplasts of BSMV_γ0_ and BSMV*_TaDJ‐1_* plants and performed western blotting with anti‐His. Results indicated that TaSnRK1α‐His was only detected in chloroplast proteins from BSMV_γ0_ plants (Figure 3F), implying that silencing of *TaDJ‐1* possibly hindered the bridge for moving TaSnRK1α−1A to the chloroplast. Therefore, we hypothesized that TaSnRK1α−1A, with the assistance of molecular chaperone TaDJ‐1, moved to the chloroplast to interact with TaPAP6L‐2B.

### 2.6 TaSnRK1α−1A Phosphorylated TaPAP6L‐2B and Improved the Protein Level of TaPAP6L‐2B In Vivo

**Figure 4:**
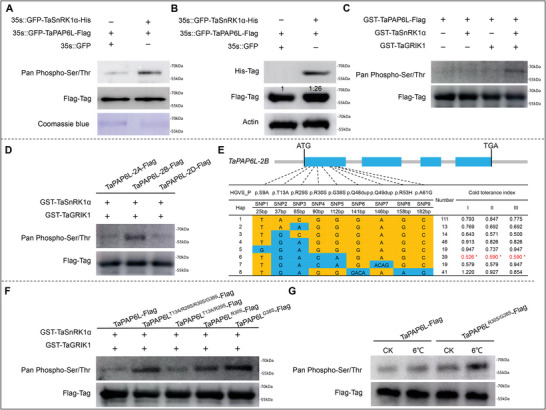
TaSnRK1α phosphorylated TaPAP6L in vivo and in vitro. A) TaSnRK1α−1A significantly improved the phosphorylation level of TaPAP6L‐2B. Immunoblots detected phosphorylation levels of TaPAP6L‐2B together with (+) or without (–) TaSnRK1α−1A in tobacco leaves cells. B) TaSnRK1α−1A significantly improved the protein abundance of TaPAP6L‐2B in tobacco leaves cells. Immunoblots detected transient expression of TaSnRK1α−1A together with (+) or without (–) TaPAP6L‐2B in tobacco leaves cells. Anti‐actin was used for loading controls. C) TaSnRK1α−1A phosphorylated TaPAP6L‐2B in *E. coli* cells. The phosphorylation status of *E. coli*‐produced TaPAP6L‐2B incubated with *E coli*‐produced TaSnRK1α−1A or/and TaGRIK1 or alone were determined by immunoblots, respectively. D) TaPAP6L‐2B had the highest phosphorylation level than its homologous proteins TaPAP6L‐2B and TaPAP6L‐2D. The phosphorylation status of *E. coli*‐produced TaPAP6L‐2B homologous proteins incubated with *E. coli*‐produced TaSnRK1α−1A and TaGRIK1 were determined by immunoblots. E) Gene structure and haplotype analysis of TaPAP6L‐2B, and comparison of the cold tolerance index among 8 haplotypes in multiple environments. I: Mar. 2018, Yuanyang; II: Feb. 2020, Yuanyang and III: Mar. 2020, Yuanyang. Statistical significance was determined by a two‐sided *t*‐test (* *P* < 0.05). F) Changes of R30S and G38S significantly enhanced the phosphorylation of TaPAP6L‐2B mediated by TaSnRK1α−1A in *E. coli* cells. The phosphorylation status of *E. coli*‐produced wild type and mutants of TaPAP6L‐2B incubated with *E coli*‐produced TaSnRK1α−1A and TaGRIK1 were determined by immunoblots. G) Changes in R30S and G38S significantly enhanced TaPAP6L‐2B phosphorylation in wheat protoplasts affected by low temperature.

As a Ser/Thr protein kinase, SnRK1 mainly functions by phosphorylating serine and threonine residues of substrate proteins.^[^ ^46^, ^47^, ^48^ ^]^ To explore whether TaSnRK1α−1A could phosphorylate TaPAP6L‐2B, we transfected proteins of tobacco leaf cells with 35S::GFP‐TaSnRK1α‐His and 35S::GFP‐TaPAP6L‐Flag. Immunoprecipitation with anti‐Flag antibody, and detection by immunoblots with Pan anti‐phosphorylation and anti‐Flag antibody showed that the addition of TaSnRK1α−1A significantly improved the phosphorylation level of TaPAP6L‐2B (**Figure** **4**A), suggesting that TaPAP6L‐2B was an endogenous substrate of TaSnRK1α−1A.

To further illustrate whether TaSnRK1α−1A could affect the protein level of TaPAP6L‐2B, we detected proteins in tobacco leaf cells transfected with 35S::GFP‐TaSnRK1α‐His and 35S::GFP‐TaPAP6L‐Flag by immunoblots with anti‐Flag antibody. Results revealed that the addition of TaSnRK1α−1A significantly improved the protein abundance of TaPAP6L‐2B (Figure 4B). These results suggested that TaSnRK1α−1A phosphorylates TaPAP6L‐2B and promotes TaPAP6L‐2B protein accumulation.

### 2.7 TaSnRK1α−1A Phosphorylated TaPAP6L‐2B In Vitro

To further confirm whether TaSnRK1α−1A directly phosphorylates TaPAP6L‐2B, we performed in vitro phosphorylation experiments. Previous studies have reported that total extraction from TaSnRK1α−1A could not directly phosphorylate substrates in vitro, but TaGRIK1 (TraesCS4D02G045900, Serine/threonine‐protein kinase) kinase was able to activate the activity of TaSnRK1α to exercise kinase function.^[^ ^49^, ^50^, ^51^ ^]^ Therefore, we added TaGRIK1 to TaSnRK1α−1A to detect the phosphorylation level of TaPAP6L‐2B. Results indicated that either individual TaSnRK1α−1A or individual TaGRIK1 did not phosphorylate TaPAP6L‐2B, however, TaSnRK1α−1A with the addition of TaGRIK1 significantly phosphorylated TaPAP6L‐2B (Figure 4C).

Blast analysis in the genome of Chinese Spring showed that *TaPAP6L* has three homologous genes *TaPAP6L‐2A* (*TraesCS2A02G145900)* on 2A, *TaPAP6L‐2B* (*TraesCS2B02G171300*) on 2B, and *TaPAP6L‐2D* (*TraesCS2D02G150500*) on 2D. To identify which one plays a more important role, we cloned sequences of the three homologous genes from Chinese Spring and purified the corresponding proteins TaPAP6L‐2A, TaPAP6L‐2B, and TaPAP6L‐2D by prokaryotic expression. In vitro phosphorylation results showed that TaPAP6L‐2B had the significantly highest phosphorylation level among the three proteins (Figure 4D). Therefore, we assumed that TaPAP6L‐2B possibly played a relatively more important role in regulating wheat cold stress among three homologous proteins.

### 2.8 Two Phosphorylation Sites R30S and G38S in TaPAP6L‐2B Possibly Affected Wheat Cold Tolerance

To excavate superior haplotypes, we sequenced *TaPAP6L‐2B* in the collected 480 wheat varieties containing the association panel and obtained 9 polymorphism sites forming 8 main haplotypes in coding region of *TaPAP6L‐2B*. Association analysis of *TaPAP6L‐2B* haplotypes with the cold tolerance index (CTI) showed that varieties with *TaPAP6L‐2B*_Hap6 and *TaPAP6L‐2B*_Hap7 possessed relatively stronger cold tolerance among 8 haplotypes over two years. Compared with the most frequent haplotype *TaPAP6L‐2B*_Hap1, *TaPAP6L‐2B*_Hap6, and *TaPAP6L‐2B*_Hap7 possessed four deduced amino acid changes of T13A, R29S, R30S, and G38S (Figure 4E). To identify which sites in TaPAP6L‐2B were significantly phosphorylated by TaSnRK1α−1A, we successfully cloned three cDNA sequences of *TaPAP6L‐2B*_Hap1 from Chinese Spring, *TaPAP6L‐2B_Hap6* (*TaPAP6L^T13A/R29S/R30S/G38S^*) from Keyu 368 and TaPAP6L‐2B_Hap4 (*TaPAP6L^T13A/R29S^*) from Xinong 18. Based on *TaPAP6L*_Hap1, we cloned cDNA sequences of *TaPAP6L^R30S^* and *TaPAP6L^G38S^* using a rapid site‐directed mutagenesis kit, respectively. Subsequently, we successfully in vitro purified these five corresponding proteins (TaPAP6L, TaPAP6L^T13A/R29S/R30S/G38S^, TaPAP6L^T13A/R29S^, TaPAP6L^R30S^, and TaPAP6L^G38S^). In vitro differential phosphorylation assay using TaSnRK1α−1A as a kinase showed that the phosphorylation levels of TaPAP6L^T13A/R29S/R30S/G38S^, TaPAP6L^R30S^ and TaPAP6L^G38S^ were significantly increased, but the phosphorylation level of TaPAP6L^T13A/R29S^ had no significant difference compared with TaPAP6L‐2B (Figure 4F), implying that changes of R30S and G38S significantly enhanced the phosphorylation of TaPAP6L‐2B. These results suggested that *TaPAP6L‐2B_Hap6* possessed relatively stronger tolerance, possibly due to the generation of two phosphorylation sties R30S and G38S.

Next, we transiently overexpressed *TaPAP6L‐2B* and *TaPAP6L‐2B^R30S/G38S^* in protoplasts of wheat leaves before and after cold treatment. TaPAP6L‐2B proteins from wheat protoplasts were immunoprecipitated with anti‐Flag antibody and were detected by immunoblots. Results showed that cold stress increased the phosphorylation level of TaPAP6L, and visibly affected the phosphorylation level of TaPAP6L^R30S/G38S^ (Figure 4G), implying that R30S and G38S as keys phosphorylation site of TaPAP6L‐2B is response to cold stress.

### 2.9 EMS Mutants and Overexpression Verified *TaPAP6L* Positively Modulating Wheat Cold Stress

**Figure 5:**
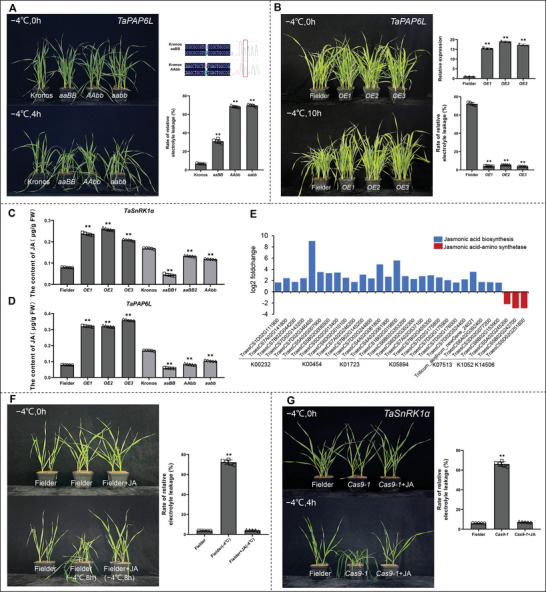
*TaPAP6L* positively modulated wheat cold stress possibly due to the accumulation of JA. A) Mutation sites of *TaPAP6L* in Kronos EMS mutants and a comparison of phenotype and physiological indices of mutants and the controls (*n* = 5) under cold stress. B) The relative expressions of *TaPAP6L‐2B* in *TaPAP6L‐2B‐OE* lines (*n* = 3) and a comparison of phenotype and physiological indices of *TaPAP6L‐2B‐OE* lines and control (*n* = 5) under cold stress. C) JA (Jasmonic acid) was measured in *TaSnRK1α−1A‐OE* lines and EMS mutants (*n* = 5); D) JA was measured in *TaPAP6L‐2B‐OE* lines and EMS mutants (*n* = 5). E) A total of 27 DEGs were involved in the synthesis of *α*‐linolenic acid identified by transcriptome sequencing. F) Exogenous JA (100 µm) significantly enhanced wheat (*n* = 5) cold tolerance. The relative electrolyte leakage rate and relative water content (*n* = 5) were determined. G) Exogenous JA (100 µm) significantly enhanced wheat (*n* = 5) cold tolerance in *TaSnRK1α*‐edited lines. The relative electrolyte leakage rate and relative water content (*n* = 5) were determined. Values are presented as mean ± SE. Statistical significance was determined by a two‐sided *t*‐test (* *P* < 0.05, ** *P* < 0.01).

To demonstrate the role of *TaPAP6L* in regulating wheat cold stress, we screened two mutants, aaBB (K2368) and AAbb (K4490) of *TaPAP6L* from the EMS‐mutagenized tetraploid wheat Kronos library. We then backcrossed them twice with wild‐type Kronos to get BC_2_ lines, respectively, and also crossed aaBB and AAbb mutants to obtain aabb double mutants. The BC_2_ lines and aabb mutants of *TaPAP6L* were used for cold treatment. After cold stress (−4 °C, 4 h), all three mutants (aaBB, AAbb, and aabb) showed significantly decreased cold tolerance, and possessed significantly increased relative electrolyte leakage rates compared with wild type (**Figure** **5**A).

To further verify *TaPAP6L* regulating cold stress by overexpression, we constructed an overexpression vector LGY‐OE3 containing *TaPAP6L‐2B* and then transferred it into Fielder for overexpression. We detected expression levels of positive *TaPAP6L‐2B*‐overexpression (*TaPAP6L‐2B‐OE*) lines by qRT‐PCR. Three positive lines with high expression levels were further self‐crossed into T_3_ generation and then were further detected for positive and high expression level. The T_3_ non‐segregating plants of three lines with high expression levels were selected for cold stress. After cold treatment (−4 °C, 10 h), *TaPAP6L‐2B‐OE* transgenic wheat plants showed significantly enhanced cold tolerance and possessed significantly decreased relative electrolyte leakage rates compared with wild type (Figure 5B). These results demonstrated that *TaPAP6L* positively regulated cold tolerance in common wheat.

### 2.10 *TaSnRK1α−1A* Modulated Cold Tolerance Possibly through the Interaction with TaPAP6L‐2B Mediating JA Content

Phytohormones as plant growth regulators modulate the adaptation process of plants to the environment. Many studies have reported the important role of phytohormones in abiotic stress.^[^ ^38^, ^52^, ^53^, ^54^ ^]^ To determine whether *TaSnRK1α* was associated with phytohormones, we measured four common endogenous phytohormones (abscisic acid, ABA; 3‐Indoleacetic acid, IAA; jasmonic acid, JA; salicylic acid, SA) in overexpression and mutant lines of *TaSnRK1α*. Results found that JA content was significantly increased in *TaSnRK1α−1A‐OE* wheat plants but was significantly decreased in *TaSnRK1α* EMS mutants compared with their wild types (Figure 5C; Figure S2, Supporting Information).

As a member of fibrillins, *TaPAP6L* was possibly involved in biosynthesis JA.^[^ ^55^ ^]^ To detect the influence of *TaPAP6L* on JA, we measured JA content in overexpression and mutant lines of *TaPAP6L*. The *TaPAP6L‐2B‐OE* lines had significantly higher JA, whereas *TaPAP6L* EMS mutants (aaBB, AAbb, and aabb) had lower JA compared with their wild types (Figure 5D). To illustrate possible mechanism of *TaPAP6L* regulating JA, we sequenced the transcriptomes of *TaPAP6L‐2B*‐*OE* and wild‐type plants. Results showed that 27 significantly up‐regulated differentially expressed genes (DEGs) were involved in the synthesis of *α*‐Linolenic acid which is the precursor of JA biosynthesis, whereas three DEGs (Jasmonic acid‐amino synthetases, *JAR1s*) responsible for JA degradation into jasmonoyl‐isoleucine (JA‐Ile) were significantly down‐regulated (Figure 5E; Table S3, Supporting Information).^[^ ^56^, ^57^ ^]^ These results suggested that *TaPAP6L‐2B‐OE* increased the JA content, most likely by promoting the accumulation of *α*‐Linolenic acid and inhibiting the degradation of JA.

### 2.11 Exogenous JA can Enhance Cold Tolerance of Wheat

To further strengthen the relationship between JA and cold stress in wheat plants, we sprayed exogenous JA on wild‐type Fielder and *TaSnRK1α*‐edited plants. Results showed that wild‐type Fielder plants with exogenous JA significantly improved cold tolerance and possessed significantly increased relative water content and significantly decreased relative electrolyte leakage rate after cold stress (−4 °C, 8 h) compared with controls (Figure 5F). On the other hand, *TaSnRK1α*‐edited wheat plants with exogenous JA obviously restored cold tolerance and possessed significantly increased relative water content and significantly decreased relative electrolyte leakage rate after cold stress (−4 °C, 4 h) compared with controls (Figure 5G). In addition, we sprayed exogenous JA on *TaPAP6L* EMS mutants and *TaPAP6L‐2B‐OE* plants. Results showed that *TaPAP6L* EMS mutants with exogenous JA significantly increased cold tolerance after cold stress (−4 °C, 4 h) compared with controls (Figure S3, Supporting Information), and *TaPAP6L‐2B‐OE* plants with exogenous JA showed obviously enhanced cold tolerance after cold stress (−4 °C, 14 h) compared with controls (Figure S4, Supporting Information). These results suggested that exogenous application of JA could significantly enhance the cold tolerance of wheat.

**Figure 6:**
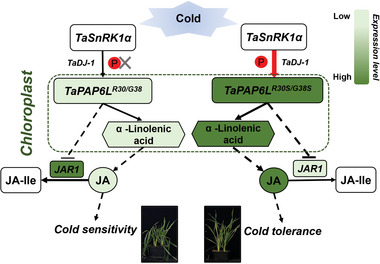
Putative model of *TaSnRK1α* regulating *TaPAP6L*‐mediated cold stress in wheat. When wheat plants were exposed to cold stress, TaSnRK1α was enriched to the surface of chloroplast with the help of TaDJ‐1. TaSnRK1α phosphorylated TaPAP6L‐2B^R30S/G38S^ and thereby caused accumulation of TaPAP6L‐2B^R30S/G38S^ to increase JA content by promoting α‐linolenic acid synthesis and inhibiting JA degradation (by down‐regulating *JAR1*), finally wheat plants with TaPAP6L‐2B‐Hap6 (TaPAP6L^R30S/G38S^) showed cold tolerance due to the phosphorylation of TaPAP6L^R30S/G38S^ from TaSnRK1α. However, wheat plants with TaPAP6L‐2B‐Hap1 (TaPAP6L^R30/G38^) showed cold sensitivity due to no obvious phosphorylation of TaPAP6L^R30/G38^ from TaSnRK1α.

In conclusion, cold stress resulted in the enrichment of TaSnRK1α at the chloroplast under the guidance of TaDJ‐1, where it facilitated the accumulation of TaPAP6L protein through phosphorylation. TaPAP6L increased JA content by promoting α‐linolenic acid synthesis and inhibiting JA degradation, thereby enhancing cold tolerance in wheat plants (**Figure** **6**).

## 3 Discussion

As one of the most important food crops, wheat is widely planted all over the world. However, cold stress seriously threatens wheat growth and yield. Frost events led to $360 million of yield losses annually in the Australian wheat production.^[^ ^58^ ^]^ Cold stress can significantly reduce the live leaf area and soluble carbohydrate accumulation and ultimately negatively affect yield.^[^ ^59^ ^]^ In the vegetative stage of wheat, cold stress can lead to leaf wilt, which is not conducive to wheat growth and development.^[^ ^60^ ^]^ Developing cold‐tolerant wheat germplasm for wheat breeding by pyramiding multiple superior genes is an effective strategy to alleviate the damage of cold stress in wheat production. In the present study, we identified two important cold stress genes, *TaSnRK1α* and *TaPAP6L*, that could be potentially used for pyramiding breeding to improve cold tolerance in wheat breeding program.

As a member of evolutionarily conserved kinase family, SnRK1 is divided into three subfamilies (SnRK1, SnRK2, and SnRK3) in plants.^[^ ^61^, ^62^ ^]^ SnRK1 is a heterotrimeric complex containing *α* subunit with catalytic activity, *β* subunit with regulatory function and *γ* subunit with anchoring function, and its function is mainly determined by the *α* subunit.^[^ ^63^, ^64^ ^]^ In Arabidopsis, *SnRK1α* is involved in growth regulation, cell proliferation, and energy metabolism.^[^ ^65^, ^66^, ^67^ ^]^ In S. lycopersicum, *SnRK1α* is involved in the immunity of biviridae.^[^ ^68^ ^]^ In rice, *OsSnRK1a* positively modulated salicylic acid and enhanced JA‐mediated defense responses after inoculation with *M. grisea*.^[^ ^69^ ^]^ The SnRK1 kinases control metabolism, growth, and development, as well as stress tolerance, by direct phosphorylation of metabolic enzymes and regulatory proteins and by extensive transcriptional regulation.^[^ ^70^ ^]^ In Arabidopsis, SnRK1 phosphorylation of FUSCA3 positively regulates embryogenesis, seed yield, and plant growth at high temperature.^[^ ^71^ ^]^ In rice, the *OsNAC23‐Tre6P‐SnRK1a* feed‐forward loop regulates sugar homeostasis and grain yield.^[^ ^47^ ^]^ SnRK1 phosphorylation of AL2 delays cabbage leaf curl virus infection,^[^ ^72^ ^]^ and SnRK1 regulates chromatin‐associated OXS3 family proteins localization through phosphorylation.^[^ ^73^ ^]^ SnRK1α‐mediated phosphorylation of a cytosolic ATPase positively regulates rice innate immunity and is inhibited by *U. virens* effector SCRE1.^[^ ^48^ ^]^ Compared with many plant species (i.e., Arabidopsis and rice etc.), hexaploid wheat possessed a higher number of *SnRK* genes. Genome‐wide identification and expression analysis of *SnRK* gene in wheat showed that the *SnRK* gene family plays an important role in the regulation of abiotic stress and different pathways.^[^ ^74^ ^]^ In wheat, *SnRK1* was reported to be involved in seed germination and fusarium head blight resistance.^[^ ^4^, ^75^ ^]^ Up to date, study of *SnRK1α* in wheat has mainly focused on growth and development as well as disease resistance, while the related research on *SnRK1* on abiotic stress has been less reported. In this study, the potential of *TaSnRK1α* to regulate wheat cold tolerance was successfully explored through multiple molecular techniques. We revealed that TaSnRK1α phosphorylated TaPAP6L and mediated endogenous JA content to regulate cold tolerance, and this regulation was involved in molecular chaperone TaDJ‐1 due to different sub‐cellular localizations of TaSnRK1α and TaPAP6L. Therefore, we brought forth a new insight into dissecting molecular mechanism of TaSnRK1α modulating wheat cold tolerance.

FBNs (Fibrillins) are a large protein family in photosynthetic organisms.^[^ ^76^ ^]^ Members of the FBN family in the thylakoid and stroma have been reported to be associated with multiple environment stresses through involving in the storage, transport, and synthesis of lipid molecules.^[^ ^77^ ^]^ As a member of FBN family, PAP6 is a homologue of FBN4 in Arabidopsis,^[^ ^78^ ^]^ and was reported to regulate the expression of genes involved in cytokinins synthesis in Arabidopsis.^[^ ^79^ ^]^ A recent study indicated that *TaPAP6L* modulated JA‐mediated grain size in wheat,^[^ ^80^ ^]^ and may play an important role in wheat cold tolerance by VIGS.^[^ ^17^ ^]^ In this study, we investigated a significant increase of JA content in *TaSnRK1α−1A‐OE* transgenic wheat lines and screened an interaction protein TaPAP6L‐2B that was possibly involved in JA synthesis. We further verified TaSnRK1α−1A interacting and phosphorylating TaPAP6L‐2B and revealed important phosphorylation sites and superior haplotypes of TaPAP6L‐2B. Therefore, our study further elucidates the regulatory mechanism of TaPAP6L as a member of the FBN family in cold tolerance.

Jasmonic acid (JA), a group of oxidized phospholipid compounds ubiquitous in the plant kingdom, is a key signal regulating various plant processes. The lipid‐derived phytohormone JA regulates plant growth, development, secondary metabolism, resistance to insect attack and pathogen infection, tolerance to abiotic stresses, etc.^[^ ^81^ ^]^ MYC2/JIN1 protein is involved in JA signal transduction and plant adaptation to salt stress, and JA impairs salt tolerance in Arabidopsis seedlings through *MYC2*‐mediated inhibition of Catalase 2 () expression.^[^ ^82^ ^]^ Recently, JA is reported to be involved in the regulation of root development under drought stress by promoting xylem differentiation from procambial cells in roots of Arabidopsis.^[^ ^83^ ^]^ The regulatory effect of JA under salt stress is correlated with the increase in JA level, thus improving ion transport, osmotic regulation, and antioxidant defense.^[^ ^84^ ^]^ JA was also reported to be involved in tolerance to cold stress. JA positively regulates the transcription pathway of CBF to up‐regulate downstream cold response genes, and ultimately improve plant cold tolerance; meanwhile, JA interacts with other hormone signaling pathways (such as auxin, ethylene, and gibberellic acid) to regulate leaf aging and cold stress.^[^ ^38^ ^]^ In this study, *TaPAP6L‐2B* significantly up‐regulated 27 differentially DEGs involved in the synthesis of *α*‐Linolenic acid but down‐regulated 3 DEGs that were responsible for JA degradation. We further revealed that upregulation of *TaPAP6L‐2B* significantly increased JA content in overexpression lines under cold stress. Moreover, exogenous JA application significantly enhanced wheat cold tolerance. These results indicated that *TaSnRK1α* modulates *TaPAP6L*‐mediated wheat cold tolerance possibly through regulating endogenous JA contents.

In summary, we found that *TaSnRK1α*‐edited lines and EMS mutants showed significantly reduced cold tolerance and JA content in wheat plants but *TaSnRK1α−1A−OE* lines showed significantly increased cold tolerance and JA content. Therefore, we assumed that *TaSnRK1α* regulated wheat cold tolerance by mediating endogenous JA content. Exogenous JA significantly enhanced cold tolerance in *TaSnRK1α*‐edited wheat lines and wild type. Subsequently, we found that overexpression of TaPAP6L‐2B which is a phosphorylation substrate of TaSnRK1α−1A also significantly increased cold tolerance and JA content in wheat plants. These results suggested that *TaSnRK1α−1A* altered endogenous JA content, possibly through phosphorylating TaPAP6L‐2B, and thereby enhanced wheat cold tolerance (Figure 6). Therefore, the relationship among *TaSnRK1α*, *TaPAP6L*, and JA could be used to construct a regulatory network between phytohormones and cold stress for potential application of wheat cold tolerance breeding.

## 4 Experimental Section

### Growth Conditions and Cold Stress

Wheat seedlings were grown in an illuminated incubator at 23/18 °C day/night temperatures under 16/8 h light/dark photoperiod and 500 mol m^−2^ s^−1^ light intensity. The cold treatments were performed according to the method as previously reported with minor modification.^[^ ^17^ ^]^ After 3–4 weeks, the seedlings were treated with cold stress. All plants with Fielder background were exposed to cold stress at −4 °C from 4 to 14 h. All plants with Kronos background were exposed to cold stress at −4 °C for 4 h. All plants with Zhengmai9023 background were exposed to cold stress at −4 °C for 12 h.

Jasmonic acid (JA) pretreatment was performed according to methods described previously with minor modification.^[^ ^85^ ^]^ Wheat seedlings at three‐leaf stage of Fielder, CRISPR‐Cas9–mediated lines of *TaSnRK1α* (*TraesCS1A02G350500*, sucrose non‐fermenting‐1‐related protein kinase alpha subunit), EMS (Ethyl methanesulfonate) mutants, and overexpressed (*TaPAP6L‐2B‐OE*) lines of *TaPAP6L* (*TraesCS2B02G171300*, plastid‐lipid‐associated protein 6, chloroplastic‐like), were spray with either 100 µm JA or an equal amount of distilled water (control) for 5 consecutive days, respectively. All plants with Fielder background were exposed to cold stress at −4 °C from 4 to 14 h. All plants with Kronos background were exposed to cold stress at −4 °C for 4 h.

### Population Materials and Cold Tolerance Index

The wheat association panel, consisting of 243 wheat accessions, as we previously reported was planted at Yuanyang (35.04°N, 113.94°E) in 2017–2018 and 2019–2020 cropping seasons, and at Zhengzhou (34.87°N, 113.60°E) in 2017–2018 cropping season.^[^ ^80^, ^86^ ^]^ The F_10_ RIL population (SC) containing 166 lines, developed from the cross of Shanghai3/Catbird×Naxos, was planted at Zhengzhou in 2015–2016 cropping season and at Yuanyang in 2017–2018 cropping season. No drought stresses occurred in field. The cold tolerance index of all surveyed accessions was investigated in February and March of each year and classified into four ranks (3, 2, 1, 0) from high to low according to the standards of the Wheat Cultivar Approval Committee of the Yellow and Huang wheat regions (i.e., sensitive, moderately sensitive, moderately tolerant, and cold tolerant, respectively) as previously described.^[^ ^17^ ^]^

### Genome‐Wide Association Study (GWAS)

All 243 wheat accessions were genotyped using the Wheat 660K SNP array as we described previously.^[^ ^80^, ^86^ ^]^ The PCA analysis of the Panel was showed in our previous study.^[^ ^87^ ^]^ After quality control, only SNPs with minor allele frequency (MAF) > 0.05 and missing data < 20% in the association panel were kept for GWAS analysis using PLINK software.^[^ ^88^ ^]^ The threshold (1.0E‐3) for *P*‐value was calculated using a modified Bonferroni correction method.^[^ ^89^ ^]^ Haplotype analysis was performed using Haploview v4.2.

### Bulked Segregant RNA‐Seq Analysis in the F10 RIL Population

The F_10_ RIL population (SC) displayed clear segregation of cold tolerance based on the investigation of classification for cold tolerance (from 0 to 3) in the field. The leaves of 3 cold‐tolerant pools (CTP) (cold stress for 0 h and 24 h, respectively) and 3 cold‐sensitive pools (CSP) were collected under cold stress for 0 h, 24 h, and 48 h at the three‐leaf stage in the greenhouse, respectively. Each cold‐tolerant or cold‐sensitive pool was composed of an equivalent mixture of leaves from 10 lines of the RIL population with level 0 or 3 under the 4 environments. Sampled leaves were rapidly frozen in liquid nitrogen and stored at −80 °C for bulked segregant RNA sequencing (BSR‐Seq). In addition, *TaPAP6L‐2B‐OE* lines and Fielder were sampled for transcriptome analysis.

The RNA concentration was measured using NanoDrop 2000 (Thermo Fisher Scientific, Wilmington, DE). RNA integrity was assessed using the RNA Nano 6000 Assay Kit of the Agilent Bioanalyzer 2100 system (Agilent Technologies, CA, USA). The clustering of the index‐coded samples was performed on a cBot Cluster Generation System using TruSeq PE Cluster Kit v4‐cBot‐HS (Illumia) according to the manufacturer's instructions. Approximately 11G clean reads were generated for each sample. Bowtie2v2.2.3 was used to map clean reads to the genome of Chinese Spring (IWGS Ref Seqv1.0) to obtain the location information of these genes. The difference expression genes (DEGs) were detected using the DESeq1.18.0 R package functions estimate SizeFactors and nbinomTest. The threshold for significantly differentially expressed genes was set as *P* value < 0.05 and fold change > 2. Results of bulked segregant RNA‐seq (BSR) analysis of the F_10_ RIL population (SC) in a 9‐Mb (532‐541 Mb) interval on 1A are shown in Table S1 (Supporting Information).

### Virus‐Induced Gene Silencing (VIGS)

The cold sensitive wheat cultivar Zhengmai 9023 was used for virus‐induced gene silencing (VIGS) using infection of barley stripe mosaic virus (BSMV) vector. Primers (Table S4, Supporting Information) of candidate genes were designed using Primer 3.0, and 237‐bp, 194‐bp, and 181‐bp fragments were generated for *TraesCS1A02G350500*, *TraesCS1A02G360300*, and *TraesCS1A02G360400*, respectively. The *α*, *β*, and *γ* RNAs of the BSMV genome were synthesized from linearized plasmids, using Ribo MAXTM Large Scale RNA Production System‐T7 (Promega, Madison). The original BSMV_0_ was constructed from *α, β*, and γ RNAs derived from the original empty pSL038–1 vector and served as the viral control. The plants inoculated with BSMV:PDS (phytoene desaturase) were used as the positive control. BSMV*_TaPDS_* (GenBank: FJ517553.1), as mentioned by Zhang et al. (2016) was used to monitor the time course of VIGS as a positive control. Transcripts of each vector (*α, β, γ*, or recombinant *γ*‐gene) were mixed in a 1:1:1 ratio to prepare for inoculating wheat plants. The mixture RNA virus was added to the FES buffer to inoculate onto the second leaves of the silenced seedlings, and after darkness for 24 h, all plants were grown at a condition of 23–25 °C with 60–80% relative humidity. After two weeks, the third and fourth leaf tissues were collected to measure the physiological index. qRT‐PCR was performed to determine the efficiency of silencing using primers in Table S4 (Supporting Information).

### qRT‐PCR Analysis

Total RNA was extracted from different materials using total RNA kits (TaKaRa, Dalian, China). Two‐step PrimeScript^TM^ RT reagent kit and gDNA Eraser were used for RT reaction by the Bio‐Rad CFX96 Real‐Time PCR system. The *β*‐actin gene (No. AB181991) was an endogenous control. Relative expression levels were evaluated according to the relative quantification method (2^−ΔΔCT^).^[^ ^90^ ^]^

### EMS Mutants of TaSnRK1α and TaPAP6L Genes

Seeds of the tetraploid wheat Kronos were mutated by the team of Jorge Dubcovsky from the University of California, Davis, using the chemical mutagen methanesulfonic acid (EMS). Two *TaSnRK1a‐1A* (from 1A chromosome) mutants Kronos331 (G/A, stop gained) and Kronos4218 (G/A, splice_acceptor_variant), *TaSnRK1a‐1B* (from 1B chromosome) mutant Kronos4220 (C/T, missense variant); *TaPAP6L‐2A* (from 2A chromosome) mutant Kronos2368 (G/A, stop gained), and *TaPAP6L‐2B* (from 2B chromosome) mutant Kronos4490 (G/A, splice_donor_variant) were used to backcross twice with Kronos to generate BC_2_ lines, respectively. We crossed Kronos2368 and Kronos4490 to obtain aabb (*TaPAP6L*) double mutants.

### Overexpression of the TaSnRK1α−1A Gene and TaPAP6L‐2B Gene

To produce *TaSnRK1α−1A‐OE* and *TaPAP6L‐2B*‐*OE* plants, the entire coding sequences (CDSs) of *TaSnRK1α−1A* and *TaPAP6L‐2B* were cloned and linked into the LGY‐OE3 vector with the Ubi promoter, respectively. These vectors containing targeted genes were transformed by *Agrobacterium*‐mediated infection into immature embryos of hexaploid wheat cultivar Fielder to obtain the *TaSnRK1α−1A‐OE* and *TaPAP6L‐2B‐OE* transgenic lines. Positively T_0_ transgenic plants detected by PCR were self‐pollinated into T_3_ generations. Three independent lines of T_3_ plants with high expression levels by qRT‐PCR were used for further analysis.

### CRISPR/Cas9 Gene Editing of TaSnRK1α

The target sequences were designed and selected using CRISPRdirect (http://crispr.dbcls.jp/) and CRISPOR (http://crispor.tefor.net/) to minimize off‐target effects. Two sgRNAs of *TaSnRK1α* were designed based on homology searches against the genome of Chinese Spring. The recombinant plasmid was generated by introducing the guide sgRNA_1_ and sgRNA_2_ into the binary vector pBUE411 that is modified with the wheat TaU3 promoter and guides the RNA scaffold. The constructed vector was introduced into the *A. tumefaciens* strain EHA105 and then transferred into Fielder for genetic transformation. *TaSnRK1α*‐edited plants were self‐pollinated into T_2_ generations. *TaSnRK1α*‐edited plants were sequenced to detect the targeted mutation by Hi‐Tom and Sanger sequencing (Table S2, Supporting Information).^[^ ^16^ ^]^

### Measurement of Phytohormones and Physiological Indexes

*TaSnRK1α−1A‐OE* lines and EMS mutants were used to determine the phytohormones, including Abscisic acid (ABA), 3‐Indoleacetic acid (IAA), Jasmonic acid (JA) and Salicylic acid (SA) based on the liquid chromatography analysis method according to previous reports.^[^ ^91^ ^]^ Besides, the JA content in *TaPAP6L‐2B‐OE* lines and EMS mutants were also determined. In order to assess the effect of cold stress, relative water content and relative electrolyte leakage rate were measured using fresh wheat seedlings.

### Yeast Two‐Hybrid (Y2‐H) Assays

The CDS sequence of *TaSnRK1α−1A* was recombined to the vector pGBKT7. The cold‐stressed wheat seedling of Ak58 was used to construct a cDNA library for screening interaction proteins using yeast two‐hybrid (Y2‐H) assay following the manufacturer's protocol (Clontech, USA). The yeast colonies that grew on SD/‐Trp‐Leu‐His‐Ade and showed *β*‐galactosidase activity were selected as putative interaction colonies. After sequencing, the client genes were identified. Then the cDNAs of *TaPAP6L‐2B* and *TaDJ‐1‐7B* (*TraesCS7A02G446200*, DJ‐1 family protein) were amplified and cloned into the pGADT7 vector. The bait and prey vectors were co‐transformed into yeast strain AH109 and screened on SD/‐Leu‐Trp and SD/‐Trp‐Leu‐His‐Ade to verify their interactions with *TaSnRK1α−1A*.

### Firefly Complementation Assay

To investigate the interaction between *TaSnRK1α−1A* and *TaPAP6L‐2B*, the CDS of *TaSnRK1α−1A* was inserted into pCAMBIA1300‐cLUC vector, while the CDS of *TaPAP6L‐2B* was inserted into pCAMBIA1300‐nLUC vector. The recombinant vectors were transformed into *A. strain* GV3101 and then different vector combinations were co‐transfected into lower epidermis of the *N. benthamiana* leaves. TaPAP6L‐nLUC and cLUC, nLUC and TaSnRK1α‐cLUC, cLUC and nLUC were used as negative controls. After 24 h dark and 24 h light, the co‐infiltrated leaves were observed for luciferase activity using a plant living imaging system (Berthold, Night Shade LB 985).

### Pull‐Down Assay

TaSnRK1α‐His and TaPAP6L‐GST or GST protein was expressed in *Escherichia coli* (*E*. *coli*). TaPAP6L‐GST or GST proteins were purified with buffer (50 mm Tris, 150 mm NaCl, 10 mm GSH, pH8.0) using beyoGold^TM^ GST‐tag Purification Resin according to the instruction of a GST‐tag Protein Purification Kit (Beyotime, China, Catalog No. P2262), then the pulled down proteins mixed with the SDS sample buffer. The samples were detected by immunoblot using an anti‐GST antibody (Abmart, M20007) and anti‐His antibody (Abmart, M20001).

### Protoplast Isolation and Transformation

Protoplast isolation and transactivation assays were performed as previously described by Fujii et al. (2009). Two‐week old wheat seedling (23/20 °C day/night, 10/14 h light/dark, and 500 mol m^−2^ s^−1^ light intensity) were used for protoplast isolation. Strips of young rosette leaves were treated with enzyme solution containing Cellulase R‐10 (Yakult Pharmaceutical Industry) and Macerozyme R‐10 (Yakult Pharmaceutical Industry) in the dark. After being diluted with equal volume of W5 solution (2 mm MES, pH 5.7, 154 mm NaCl, 125 mm CaCl_2_, and 5 mm KCl), the protoplasts were filtered through a nylon mesh and pelleted at 100 g for 2 min. Protoplasts were resuspended in W5 solution and kept for 30 min. Protoplasts (100 µL) in MMG solution (4 mm MES, pH 5.7, 0.4 m mannitol, and 15 mm MgCl_2_) were mixed with the plasmid mix, and incubated for 5 min with 110 µL PEG solution (40% w/v PEG‐4000, 0.2 m mannitol, and 100 mm CaCl_2_). The protoplasts were washed twice with 1 mL W5 solution, and then were cultured at room temperature for 12 h for further observation.

### Site‐Directed Mutagenesis and Expression

The site‐directed mutagenesis of R30 (R30S, AGG‐AGC) and G38 (G38S, GGC‐AGC) within the Ubi::GFP‐TaPAP6L‐Flag plasmid was performed using the Fast Site‐Directed Mutagenesis Kit (Tiangen, China, Catalog No. KM101).

Furthermore, Ubi::GFP‐TaPAP6L‐Flag and Ubi::GFP‐TaPAP6L^R30S/G38S^‐Flag in protoplasts of wheat leaves (Fielder) before and after cold stress (6 °C, 4 h) were transiently overexpressed. Proteins of transfected protoplast were immunoprecipitated by anti‐Flag antibody and were analyzed by immunoblots with anti‐Flag antibody (Abmart, M20008) and Pan‐Phospho‐Ser/Thr antibody (Abmart, T91067), respectively.

### Subcellular Localization and Bimolecular Fluorescence Complementation (BiFC) Analysis

For subcellular localization, CDSs of TaSnRK1α−1A and TaPAP6L‐2B were cloned into the pJIT163‐hGFP vector to generate Ubi::GFP‐TaSnRK1α and Ubi::GFP‐TaPAP6L, respectively. The resulting constructs were transformed into wheat protoplasts via polyethylene glycol (PEG)‐mediated transformation,^[^ ^92^ ^]^ respectively. After incubation for 20 h in the dark at 22 °C, confocal microscopy (Carl Zeiss, LSM710) was used to examine the GFP fluorescence. For bimolecular fluorescence complementation (BiFC), the CDSs of TaSnRK1α−1A and TaPAP6L‐2B were cloned into vectors pSAT1‐nEYFP‐C1 and pSAT1‐cEYFP‐C1‐B to generate nEYFP‐TaSnRK1α and cEYFP‐TaPAP6L, respectively. The resulting constructs were then co‐transformed into wheat protoplasts. After incubation for 20 h in the dark at 22 °C, confocal microscopy (Carl Zeiss, LSM710) was used to examine the YFP fluorescence.

### Chloroplast Protein Extraction of Wheat Protoplast

An appropriate amount of chloroplast extraction buffer (0.33 m Sorbitol, 50 mm HEPES‐KOH pH 8.0, 2.0 mm EDTA‐Na_2_, 2.0 mm MgCl_2_, 2.0 mm MnCl_2,_ 2.0 mm Ascorbic acid, 0.1% BSA) was added to the cultured protoplasts, resuspension protoplasts. Supernatant was taken and centrifuged at 4 °C at 1500 g for 10 min. The resulting precipitates were crude extracted protoplasts. The precipitates were suspended with 2 mL chloroplast extraction buffer solution for further use. Taking a 15 mL centrifuge tube, the bottom contains 80% Percoll (v/v) gradient solution (0.33 m Sorbitol, 50 mm HEPES‐KOH pH 8.0, 2.0 mm EDTA‐Na_2_), the upper part contains a gradient liquid covering of 30% Percoll (v/v). The prepared crude chloroplast extract was suspended on 30% Percoll and centrifuged at 7000 g for 1 h. The chloroplasts were washed two times with clean solution (0.33 m Sorbitol, 50 mm HEPES‐KOH pH 8.0, 2.0 mm EDTA‐Na_2_, 1.0 mm MgCl_2_) and were then suspended in the buffer solution. The chloroplast proteins were analyzed by sodium dodecyl sulfate‐polyacrylamide gel electrophoreses (SDS‐PAGE) with coomassie blue staining and western blotting using anti‐GFP antibody (Abmart, M20004).

### Transient Expression Assays

35S::GFP‐TaPAP6L‐Flag and 35S::GFP‐TaSnRK1α‐His or 35S::GFP were introduced into four‐week‐old tobacco (*N. benthamiana*) leaf cells by injection, and total proteins were extracted in lysis buffer (20 mm Tris‐HCl pH 8.0, 2 mm DTT, 1% Triton X‐100, 800 µm PMSF, 250 mm sucrose) containing protease inhibitor cocktail. Immunoprecipitation (IP) was carried out by incubation with 25 µL of the anti‐Flag antibody (Abmart, M20008) according to our previous study.^[^ ^93^ ^]^ A 30 µL Protein A/G PLUS‐Agarose (Santa Cruz, Lot number sc‐2003) was added, and the solution was incubated on ice for 4−6 h with gentle shaking. After incubation, the bound proteins were washed for four times with PBS buffer (137 mm NaCl, 2.7 mm KCl, 10 mm Na_2_HPO_4_, 2 mm KH_2_PO_4_ pH 7.4, 0.2 mm DTT) and were then suspended in 40 µL PBS buffer. The proteins in the buffer were analyzed by western blotting using anti‐Flag antibody (Abmart, M20008) and Pan‐Phospho‐Ser/Thr antibody (Abmart, T91067) using the ECL SuperSignal system.

To examine whether TaSnRK1α could alter the expression of TaPAP6L, protein extracts of tobacco leaves were transiently expressed with 35S::GFP‐TaPAP6L‐Flag together 35S::GFP‐TaSnRK1α‐His or 35S::GFP were analyzed by immunoblots using anti‐Flag antibody (Abmart, M20008).

### In Vitro Phosphorylation Assays

The CDS fragments of *TaPAP6L‐2B_Hap1* from Chinese Spring, *TaPAP6L‐2B_Hap6* (*TaPAP6L‐2B^T13A/R29S/R30S/G38S^*) from Keyu 368 and *TaPAP6L‐2B_Hap4* (*TaPAP6L‐2B^T13A/R29S^*) from Xinong 18 were recombined into pGEX6p‐GST vector. We performed site‐directed mutagenesis of R30 (R30S, AGG‐AGC) and G38 (G38S, GGC‐AGC) within the *pGEX6p‐GST‐TaPAP6L_Hap1‐Flag* plasmid using the Fast Site‐Directed Mutagenesis Kit (Tiangen, China, Catalog No. KM101), respectively. PCR was conducted using site‐specific primers (Table S4, Supporting Information). Positive mutants were verified by sequencing. *E. coli* BL21 (DE3) was used to express recombinant TaPAP6L‐2B, TaPAP6L‐2B^T13A/R29S/R30S/G38S^, TaPAP6L‐2B^T13A/R29S^, TaPAP6L‐2B^R30S^, and TaPAP6L‐2B^G38S^. These five proteins were purified using a GST‐tag Protein Purification Kit (Beyotime, China, Catalog No. P2262). For in vitro phosphorylation assay, 10 µg of the purified TaPAP6L‐2B, TaPAP6L‐2B^T13A/R29S/R30S/G38S^, TaPAP6L‐2B^T13A/R29S^, TaPAP6L‐2B^R30S^, or TaPAP6L‐2B^G38S^ was incubated with 5 µg TaSnRK1α−1A and 5 µg TaGRIK1 at 30 °C for 30 min in reaction buffer (20 mm Tris‐HCl pH 7.5, 5 mm MgCl_2_, 0.1 mm CaCl_2_, 50 µm ATP, and 2 mm dithiothreitol). The phosphorylation level was analyzed by western blotting using Flag‐Tag antibody (Abmart, TT0003) and Pan‐Phospho‐Ser/Thr antibody (Abmart, T91067).

## Conflict of Interest

The authors declare no conflict of interest.

## Author Contributions

L.Z. and N.Z. contributed equally to this work. F.C. conceived and designed this study. L.Z. and N.Z. cloned genes. L.Z., N.Z., S.W., and H.T. scored cold tolerance in filed. L.Z., N.Z., D.P., X. Y., L.Z., and F.C. verified the gene function and dissected molecular mechanism. L.Z., N.Z., and F.C. wrote the manuscript.

## Supporting information

Supporting Information

Supplemental Table 1

## Data Availability

The data that support the findings of this study are available in the supplementary material of this article.

## References

[1] M. A. Hassan , C. Xiang , M. Farooq , N. Muhammad , Z. Yan , X. Hui , K. Yuanyuan , A. K. Bruno , Z. Lele , L. Jincai , Frontiers in plant science 2021, 12, 676884. 34305976 10.3389/fpls.2021.676884 PMC8299469

[2] F. N. Ritonga , S. Chen , Plants (Basel, Switzerland) 2020, 9, 560. 32353940 10.3390/plants9050560 PMC7284489

[3] Y. Shi , Y. Ding , S. Yang , Plant & cell physiology 2015, 56, 7. 25189343 10.1093/pcp/pcu115

[4] Y. Song , X. Zhang , M. Li , H. Yang , D. Fu , J. Lv , Y. Ding , Z. Gong , Y. Shi , S. Yang , Journal of integrative plant biology 2021, 63, 1874. 34379362 10.1111/jipb.13161

[5] A. Ahad , A. Gul , T. S. Batool , N. U. Huda , F. Naseeer , U. Abdul Salam , M. Abdul Salam , M. Ilyas , B. Turkyilmaz Unal , M. Ozturk , Molecular biology reports 2023, 50, 6997. 37378744 10.1007/s11033-023-08584-1

[6] S. Gusain , S. Joshi , R. Joshi , Plant physiology and biochemistry : PPB 2023, 197, 107646. 36958153 10.1016/j.plaphy.2023.107646

[7] H. Li , Y. Ding , Y. Shi , X. Zhang , S. Zhang , Z. Gong , S. Yang , Dev. Cell 2017, 43, 630.e4. 29056553 10.1016/j.devcel.2017.09.025

[8] V. E. Ramirez , B. Poppenberger , Dev. Cell 2017, 43, 545. 29207256 10.1016/j.devcel.2017.10.032

[9] C. Zhao , P. Wang , T. Si , C. C. Hsu , L. Wang , O. Zayed , Z. Yu , Y. Zhu , J. Dong , W. A. Tao , J. K. Zhu , Dev. Cell 2017, 43, 618.e5. 29056551 10.1016/j.devcel.2017.09.024 PMC5716877

[10] X. Wang , X. Zhang , C. Song , Z. Gong , S. Yang , Y. Ding , The Plant cell. 2023, 35, 3585. 37279565 10.1093/plcell/koad159 PMC10473228

[11] Y. Ma , X. Dai , Y. Xu , W. Luo , X. Zheng , D. Zeng , Y. Pan , X. Lin , H. Liu , D. Zhang , J. Xiao , X. Guo , S. Xu , Y. Niu , J. Jin , H. Zhang , X. Xu , L. Li , W. Wang , Q. Qian , S. Ge , K. Chong , Cell 2015, 160, 1209. 25728666 10.1016/j.cell.2015.01.046

[12] Q. Liu , Y. Ding , Y. Shi , L. Ma , Y. Wang , C. Song , K. A. Wilkins , J. M. Davies , H. Knight , M. R. Knight , Z. Gong , Y. Guo , S. Yang , EMBO J. 2021, 40, 104559. 10.15252/embj.2020104559 PMC7809786 33372703

[13] Z. Li , B. Wang , W. Luo , Y. Xu , J. Wang , Z. Xue , Y. Niu , Z. Cheng , S. Ge , W. Zhang , J. Zhang , Q. Li , K. Chong , Sci. Adv. 2023, 9, eabq5506. 36608134 10.1126/sciadv.abq5506 PMC9821855

[14] Q. Liu , X. Zhang , Y. H. Su , X. S. Zhang , Life (Basel, Switzerland) 2022, 12, 700. 35629367 10.3390/life12050700 PMC9147279

[15] Y. Tian , K. Peng , Y. Bao , D. Zhang , J. Meng , D. Wang , X. Wang , J. Cang , Plant physiology and biochemistry : PPB 2021, 161, 86. 33581622 10.1016/j.plaphy.2021.02.005

[16] Q. Liu , C. Wang , X. Jiao , H. Zhang , L. Song , Y. Li , C. Gao , K. Wang , Science China. Life sciences. 2019, 62, 1. 30446870 10.1007/s11427-018-9402-9

[17] N. Zhang , W. Huo , L. Zhang , F. Chen , D. Cui , Molecular & cellular proteomics : MCP 2016, 15, 2954. 27402868 10.1074/mcp.M115.057232 PMC5013310

[18] N. Zhang , L. Zhang , C. Shi , L. Zhao , D. Cui , F. Chen , J. Proteome Res. 2018, 17, 2256. 29761697 10.1021/acs.jproteome.7b00745

[19] K. Xu , Y. Zhao , J. Gu , M. Zhou , L. Gao , R. X. Sun , W. W. Wang , S. H. Zhang , X. J. Yang , Plant science : an international journal of experimental plant biology 2022, 318, 111242. 35351310 10.1016/j.plantsci.2022.111242

[20] B. Kalapos , P. Dobrev , T. Nagy , P. Vítámvás , J. Györgyey , G. Kocsy , F. Marincs , G. Galiba , Plant science : an international journal of experimental plant biology 2016, 253, 86. 27969000 10.1016/j.plantsci.2016.09.017

[21] J. Yu , J. Cang , Q. Lu , B. Fan , Q. Xu , W. Li , X. Wang , Plant signaling & behavior 2020, 15, 1780403. 32619128 10.1080/15592324.2020.1780403 PMC8570709

[22] X. Li , M. Brestic , D. X. Tan , M. Zivcak , X. Zhu , S. Liu , F. Song , R. J. Reiter , F. Liu , J. Pineal Res. 2018, 64. 10.1111/jpi.12453 29149482

[23] L. Sun , X. Li , Z. Wang , Z. Sun , X. Zhu , S. Liu , F. Song , F. Liu , Y. Wang , Molecules, (Basel, Switzerland) 2018, 23, 1091. 29734723 10.3390/molecules23051091 PMC6100458

[24] M. S. Ali , K. H. Baek , Int. J. Mol. Sci. 2020, 21, 621.31963549

[25] K. Gomi , Int. J. Mol. Sci. 2021, 22, 3506.33805251

[26] J. Ruan , Y. Zhou , M. Zhou , J. Yan , M. Khurshid , W. Weng , J. Cheng , K. Zhang , Int. J. Mol. Sci. 2019, 20, 2479. 31137463 10.3390/ijms20102479 PMC6566436

[27] Y. Wang , S. Mostafa , W. Zeng , B. Jin , Int. J. Mol. Sci. 2021, 22, 8568. 34445272 10.3390/ijms22168568 PMC8395333

[28] F. Ding , X. Wang , Z. Li , M. Wang , Plants, (Basel, Switzerland) 2022, 12, 60. 36616188 10.3390/plants12010060 PMC9823970

[29] G. Langenkämper , N. Manac'h , M. Broin , S. Cuiné , N. Becuwe , M. Kuntz , P. Rey , J. Exp. Bot. 2001, 52, 1545. 11457915 10.1093/jexbot/52.360.1545

[30] I. Kim , E. H. Kim , Y. R. Choi , H. U. Kim , Plant Physiol. 2022, 189, 1363. 35404409 10.1093/plphys/kiac166 PMC9237730

[31] J. Li , J. Yang , B. Zhu , G. Xie , Plant science : an international journal of experimental plant biology 2019, 285, 230. 31203888 10.1016/j.plantsci.2019.05.007

[32] D. Torres‐Romero , Á. Gómez‐Zambrano , A. J. Serrato , M. Sahrawy , Á. Mérida , J. Exp. Bot. 2022, 73, 903. 34651644 10.1093/jxb/erab452 PMC8793873

[33] D. Laudert , U. Pfannschmidt , F. Lottspeich , H. Holländer‐Czytko , E. W. Weiler , Plant Mol. Biol. 1996, 31, 323. 8756596 10.1007/BF00021793

[34] A. Youssef , Y. Laizet , M. A. Block , E. Maréchal , J. P. Alcaraz , T. R. Larson , D. Pontier , J. Gaffé , M. Kuntz , The Plant journal : for cell and molecular biology 2010, 61, 436. 19906042 10.1111/j.1365-313X.2009.04067.x

[35] A. J. Ytterberg , J. B. Peltier , K. J. van Wijk , Plant Physiol. 2006, 140, 984. 16461379 10.1104/pp.105.076083 PMC1400577

[36] N. Repkina , A. Ignatenko , E. Holoptseva , I. Z. Miszalsk , P. Kaszycki , V. Talanova , Plants, (Basel, Switzerland) 2021, 10, 1421. 34371628 10.3390/plants10071421 PMC8309304

[37] V. V. Talanova , A. F. Titov , N. S. Repkina , A. A. Ignatenko , Doklady. Biochemistry and biophysics. 2018, 482, 238. 30397882 10.1134/S1607672918050022

[38] Y. Hu , Y. Jiang , X. Han , H. Wang , J. Pan , D. Yu , J. Exp. Bot. 2017, 68, 1361. 28201612 10.1093/jxb/erx004

[39] C. Sun , Z. Dong , L. Zhao , Y. Ren , N. Zhang , F. Chen , Plant biotechnology journal 2020, 18, 1354. 32065714 10.1111/pbi.13361 PMC7206996

[40] M. N. Danilova , N. V. Kudryakova , A. A. Andreeva , A. S. Doroshenko , E. S. Pojidaeva , V. V. Kusnetsov , Plant physiology and biochemistry : PPB 2018, 129, 90. 29852366 10.1016/j.plaphy.2018.05.023

[41] I. Amm , D. Norell , D. H. Wolf , PLoS One 2015, 10, e0140363. 26466368 10.1371/journal.pone.0140363 PMC4605529

[42] Y. Z. Deng , L. Xiao , L. Zhao , L. J. Qiu , Z. X. Ma , X. W. Xu , H. Y. Liu , T. T. Zhou , X. Y. Wang , L. Tang , H. P. Chen , Molecules, (Basel, Switzerland) 2019, 25, 71. 31878239 10.3390/molecules25010071 PMC6983240

[43] S. Hu , J. Tan , L. Qin , L. Lv , W. Yan , H. Zhang , B. Tang , C. Wang , Neurobiology of disease 2021, 160, 105527. 34626793 10.1016/j.nbd.2021.105527

[44] G. A. Jana , P. Krishnamurthy , P. P. Kumar , M. W. Yaish , Physiol. Plant. 2021, 172, 780. 33034392 10.1111/ppl.13239

[45] M. Neves , M. Grãos , S. I. Anjo , B. Manadas , Redox Biol. 2022, 51, 102283. 35303520 10.1016/j.redox.2022.102283 PMC8928136

[46] Y. Hu , J. Liu , Y. Lin , X. Xu , Y. Xia , J. Bai , Y. Yu , F. Xiao , Y. Ding , C. Ding , L. Chen , Plant Physiol. 2022, 189, 1694. 35294032 10.1093/plphys/kiac124 PMC9237689

[47] Z. Li , X. Wei , X. Tong , J. Zhao , X. Liu , H. Wang , L. Tang , Y. Shu , G. Li , Y. Wang , J. Ying , G. Jiao , H. Hu , P. Hu , J. Zhang , Mol. Plant 2022, 15, 706. 35093592 10.1016/j.molp.2022.01.016

[48] J. Yang , N. Zhang , J. Wang , A. Fang , J. Fan , D. Li , Y. Li , S. Wang , F. Cui , J. Yu , Y. Liu , W. M. Wang , Y. L. Peng , S. Y. He , W. Sun , The New phytologist 2022, 236, 1422. 36068953 10.1111/nph.18460

[49] J. D. Barajas‐Lopez , J. R. Moreno , F. M. Gamez‐Arjona , J. M. Pardo , M. Punkkinen , J. K. Zhu , F. J. Quintero , H. Fujii , The Plant journal : for cell and molecular biology 2018, 93, 107. 29094495 10.1111/tpj.13761 PMC5814739

[50] H. Jin , X. Han , Z. Wang , Y. Xie , K. Zhang , X. Zhao , L. Wang , J. Yang , H. Liu , X. Ji , L. Dong , H. Zheng , W. Hu , Y. Liu , X. Wang , X. Zhou , Y. Zhang , W. Qian , W. Zheng , Q. Shen , M. Gou , D. Wang , EMBO J. 2022, 41, 110521. 10.15252/embj.2021110521 PMC9475517 35929182

[51] Z. Zhai , J. Keereetaweep , H. Liu , R. Feil , J. E. Lunn , J. Shanklin , The Plant cell 2018, 30, 2616. 30249634 10.1105/tpc.18.00521 PMC6241258

[52] S. Saini , N. Kaur , P. K. Pati , Ecotoxicol. Environ. Saf. 2021, 223, 112578. 34352573 10.1016/j.ecoenv.2021.112578

[53] V. Verma , P. Ravindran , P. P. Kumar , BMC plant biology 2016, 16, 86. 27079791 10.1186/s12870-016-0771-y PMC4831116

[54] R. Waadt , C. A. Seller , P. K. Hsu , Y. Takahashi , S. Munemasa , J. I. Schroeder , Nature reviews. Molecular cell biology. 2022, 23, 680. 35513717 10.1038/s41580-022-00479-6 PMC9592120

[55] D. K. Singh , T. W. McNellis , Trends Plant Sci. 2011, 16, 432. 21571574 10.1016/j.tplants.2011.03.014

[56] A. J. Koo , X. Gao , A. D. Jones , G. A. Howe , The Plant journal : for cell and molecular biology 2009, 59, 974. 19473329 10.1111/j.1365-313X.2009.03924.x

[57] P. E. Staswick , I. Tiryaki , The Plant cell 2004, 16, 2117. 15258265 10.1105/tpc.104.023549 PMC519202

[58] B. E. Cheong , W. W. H. Ho , B. Biddulph , X. Wallace , T. Rathjen , T. W. T. Rupasinghe , U. Roessner , R. Dolferus , Metabolomics : Official journal of the Metabolomic Society 2019, 15, 144. 31630279 10.1007/s11306-019-1606-2 PMC6800866

[59] P. F. Li , B. L. Ma , Y. C. Xiong , W. Y. Zhang , J. Sci. Food Agric. 2017, 97, 4036. 28194804 10.1002/jsfa.8271

[60] Q. Han , G. Kang , T. Guo , Plant physiology and biochemistry : PPB 2013, 63, 236. 23298682 10.1016/j.plaphy.2012.12.002

[61] P. Coello , S. J. Hey , N. G. Halford , J. Exp. Bot. 2011, 62, 883. 20974737 10.1093/jxb/erq331

[62] L. A. Witters , B. E. Kemp , A. R. Means , Trends Biochem. Sci. 2006, 31, 13. 16356723 10.1016/j.tibs.2005.11.009

[63] R. Ghillebert , E. Swinnen , J. Wen , L. Vandesteene , M. Ramon , K. Norga , F. Rolland , J. Winderickx , The FEBS journal 2011, 278, 3978. 21883929 10.1111/j.1742-4658.2011.08315.x

[64] M. Jossier , J. P. Bouly , P. Meimoun , A. Arjmand , P. Lessard , S. Hawley , D. Grahame Hardie , M. Thomas , The Plant journal : for cell and molecular biology 2009, 59, 316. 19302419 10.1111/j.1365-313X.2009.03871.x

[65] T. Guérinier , L. Millan , P. Crozet , C. Oury , F. Rey , B. Valot , C. Mathieu , J. Vidal , M. Hodges , M. Thomas , N. Glab , The Plant journal : for cell and molecular biology 2013, 75, 515. 23617622 10.1111/tpj.12218

[66] N. G. Halford , S. Hey , D. Jhurreea , S. Laurie , R. S. McKibbin , M. Paul , Y. Zhang , J. Exp. Bot. 2003, 54, 467. 12508057 10.1093/jxb/erg038

[67] M. O'Brien , R. N. Kaplan‐Levy , T. Quon , P. G. Sappl , D. R. Smyth , J. Exp. Bot. 2015, 66, 2475. 25697797 10.1093/jxb/erv032 PMC4986862

[68] Q. Shen , M. Bao , X. Zhou , Plant signaling & behavior 2012, 7, 888. 22751295 10.4161/psb.20646 PMC3583982

[69] O. Filipe , D. De Vleesschauwer , A. Haeck , K. Demeestere , M. Höfte , Sci. Rep. 2018, 8, 3864. 29497084 10.1038/s41598-018-22101-6 PMC5832823

[70] N. Crepin , F. Rolland , Curr. Opin. Plant Biol. 2019, 51, 29. 31030062 10.1016/j.pbi.2019.03.006

[71] A. Chan , C. Carianopol , A. Y. Tsai , K. Varatharajah , R. S. Chiu , S. Gazzarrini , J. Exp. Bot. 2017, 68, 5981. 29140438 10.1093/jxb/erx379 PMC5854115

[72] W. Shen , M. B. Dallas , M. B. Goshe , L. Hanley‐Bowdoin , J. Virol. 2014, 88, 10598. 24990996 10.1128/JVI.00761-14 PMC4178870

[73] S. Xiao , L. Jiang , C. Wang , D. W. Ow , Biochem. Biophys. Res. Commun. 2020, 533, 526. 32981682 10.1016/j.bbrc.2020.08.108

[74] S. Mishra , P. Sharma , R. Singh , R. Tiwari , G. P. Singh , Sci. Rep. 2021, 11, 22477. 34795369 10.1038/s41598-021-99639-5 PMC8602265

[75] C. Jiang , R. Hei , Y. Yang , S. Zhang , Q. Wang , W. Wang , Q. Zhang , M. Yan , G. Zhu , P. Huang , H. Liu , J. R. Xu , Nat. Commun. 2020, 11, 4382. 32873802 10.1038/s41467-020-18240-y PMC7462860

[76] Y. Jiang , H. Hu , Y. Ma , J. Zhou , PeerJ 2020, 8, e9225. 32518731 10.7717/peerj.9225 PMC7258936

[77] I. Kim , H. U. Kim , J. Exp. Bot. 2022, 73, 2751. 35560204 10.1093/jxb/erac087

[78] D. K. Singh , S. N. Maximova , P. J. Jensen , B. L. Lehman , H. K. Ngugi , T. W. McNellis , Plant Physiol. 2010, 154, 1281. 20813909 10.1104/pp.110.164095 PMC2971606

[79] A. A. Andreeva , R. Vankova , I. A. Bychkov , N. V. Kudryakova , M. N. Danilova , J. Lacek , E. S. Pojidaeva , V. V. Kusnetsov , Biomolecules 2020, 10, 1658. 33322466 10.3390/biom10121658 PMC7764210

[80] M. Niaz , L. Zhang , G. Lv , H. Hu , X. Yang , Y. Cheng , Y. Zheng , B. Zhang , X. Yan , A. Htun , L. Zhao , C. Sun , N. Zhang , Y. Ren , F. Chen , Plant biotechnology journal 2023, 21, 979. 36650924 10.1111/pbi.14009 PMC10106860

[81] C. Wasternack , S. Song , J. Exp. Bot. 2017, 68, 1303. 27940470 10.1093/jxb/erw443

[82] B. Dombrecht , G. P. Xue , S. J. Sprague , J. A. Kirkegaard , J. J. Ross , J. B. Reid , G. P. Fitt , N. Sewelam , P. M. Schenk , J. M. Manners , K. Kazan , The Plant cell 2007, 19, 2225. 17616737 10.1105/tpc.106.048017 PMC1955694

[83] G. Jang , Y. D. Choi , Plant signaling & behavior 2018, 13, e1451707. 29533132 10.1080/15592324.2018.1451707 PMC5927639

[84] M. S. Sheteiwy , Z. Ulhassan , W. Qi , H. Lu , H. AbdElgawad , T. Minkina , S. Sushkova , V. D. Rajput , A. El‐Keblawy , I. Jośko , S. Sulieman , M. A. El‐Esawi , K. A. El‐Tarabily , S. F. AbuQamar , H. Yang , M. Dawood , Frontiers in plant science 2022, 13, 886862. 36061773 10.3389/fpls.2022.886862 PMC9429808

[85] M. Zhu , Y. Liu , P. Cai , X. Duan , S. Sang , Z. Qiu , Frontiers in plant science 2022, 13, 968477. 35937348 10.3389/fpls.2022.968477 PMC9355640

[86] L. Zhao , Y. Zheng , Y. Wang , S. Wang , T. Wang , C. Wang , Y. Chen , K. Zhang , N. Zhang , Z. Dong , F. Chen , Plant biotechnology journal 2023, 21, 122. 36128872 10.1111/pbi.13930 PMC9829390

[87] C. Sun , F. Zhang , X. Yan , X. Zhang , Z. Dong , D. Cui , F. Chen , Plant biotechnology journal 2017, 15, 953. 28055148 10.1111/pbi.12690 PMC5506658

[88] S. Purcell , B. Neale , K. Todd‐Brown , L. Thomas , M. A. Ferreira , D. Bender , J. Maller , P. Sklar , P. I. de Bakker , M. J. Daly , P. C. Sham , Am. J. Hum. Genet. 2007, 81, 559. 17701901 10.1086/519795 PMC1950838

[89] H. Li , Z. Peng , X. Yang , W. Wang , J. Fu , J. Wang , Y. Han , Y. Chai , T. Guo , N. Yang , J. Liu , M. L. Warburton , Y. Cheng , X. Hao , P. Zhang , J. Zhao , Y. Liu , G. Wang , J. Li , J. Yan , Nat. Genet. 2013, 45, 43. 23242369 10.1038/ng.2484

[90] K. J. Livak , T. D. Schmittgen , Methods 2001, 25, 402. 11846609 10.1006/meth.2001.1262

[91] R. Yang , T. Yang , H. Zhang , Y. Qi , Y. Xing , N. Zhang , R. Li , S. Weeda , S. Ren , B. Ouyang , Y. D. Guo , Plant physiology and biochemistry : PPB 2014, 77, 23. 24531233 10.1016/j.plaphy.2014.01.015

[92] S. D. Yoo , Y. H. Cho , J. Sheen , Nat. Protoc. 2007, 2, 1565. 17585298 10.1038/nprot.2007.199

[93] N. Zhang , L. Zhang , L. Li , J. Geng , L. Zhao , Y. Ren , Z. Dong , F. Chen , Genomics, proteomics & bioinformatics 2022, 20, 688. 10.1016/j.gpb.2020.06.008 PMC9880814 33581340

