# Supplementary material for: A TaSnRK1α Modulates TaPAP6L‐Mediated Wheat Cold Tolerance through Regulating Endogenous Jasmonic Acid
Source: Adv Sci (Weinh). 2023 Sep 22;10(31):2303478. doi: 10.1002/advs.202303478 (PMC10625090; doi:10.1002/advs.202303478)
Supplement: Supplementary file 1 — Supporting Information [file ADVS-10-2303478-s002.pdf]

ADVANCED SCIENCE
Open Access

Supporting Information

for *Adv. Sci.*, DOI 10.1002/advs.202303478

A TaSnRK1α Modulates TaPAP6L-Mediated Wheat Cold Tolerance through Regulating Endogenous Jasmonic Acid

*Lingran Zhang, Ning Zhang, Sisheng Wang, Hongyan Tian, Lu Liu, Dan Pei, Xiaodong Yu, Lei Zhao and Feng Chen**

Supporting Information

Supplemental Figure 1. VIGS (virus-induced gene silencing) experiment verified the function of *TaSnRK1α* gene.

(A-B) Phenotypes of wild type (WT), BSMV$_0$ and BSMV$_{TaSnRK1\alpha}$ plants (n = 5 plants per replicate) before and after cold stress; (C) the relative expression levels (n = 3). CK represents wheat plants without cold stress; NS represents unsilenced plants. Values are presented as mean± SE. Statistical significance was determined by a two-sided *t*-test (*: *P* < 0.05; **: *P* < 0.01).

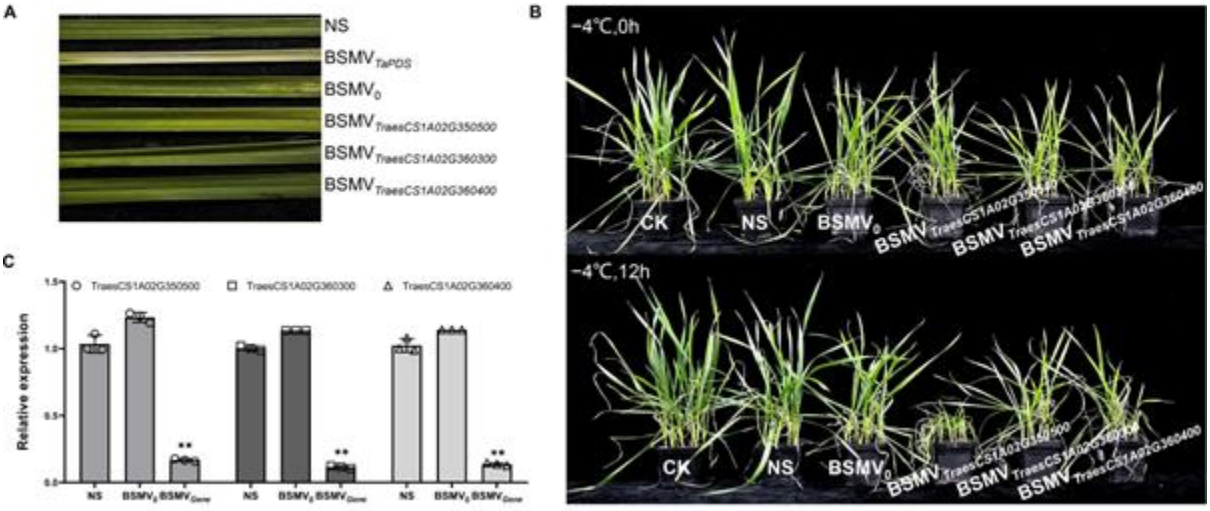

Supplementary Figure 1

Supplemental Figure 2. The contents of three endogenous phytohormones (ABA, IAA and SA) in *TaSnRK1α-1A-OE* lines and EMS mutants. The experiments were conducted with 5 plants per group; ABA: Abscisic acid; IAA: 3-Indoleacetic acid; SA: Salicylic acid. Values are presented as mean± SE. Statistical significance was determined by a two-sided *t*-test (*: *P* < 0.05; **: *P* < 0.01).

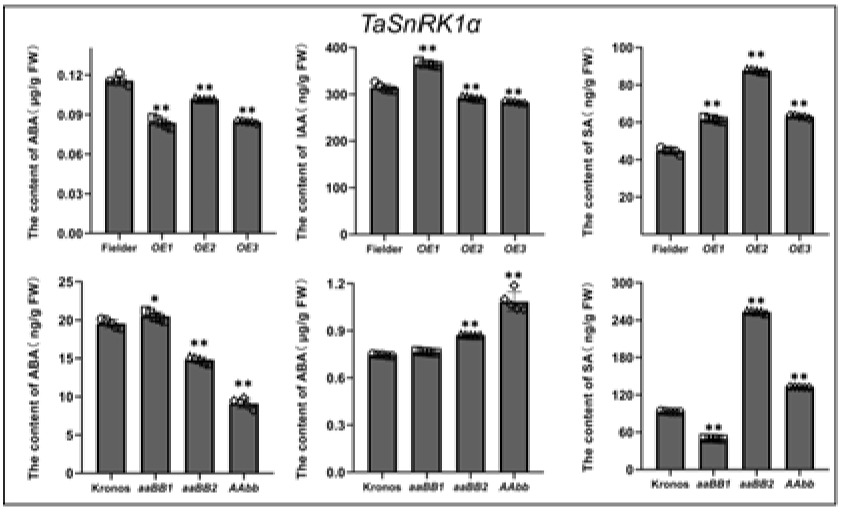

Supplementary Figure 2

Supplemental Figure 3. Exogenous JA (100 μM) significantly enhanced wheat cold tolerance in *TaPAP6L* EMS mutants after cold stress.

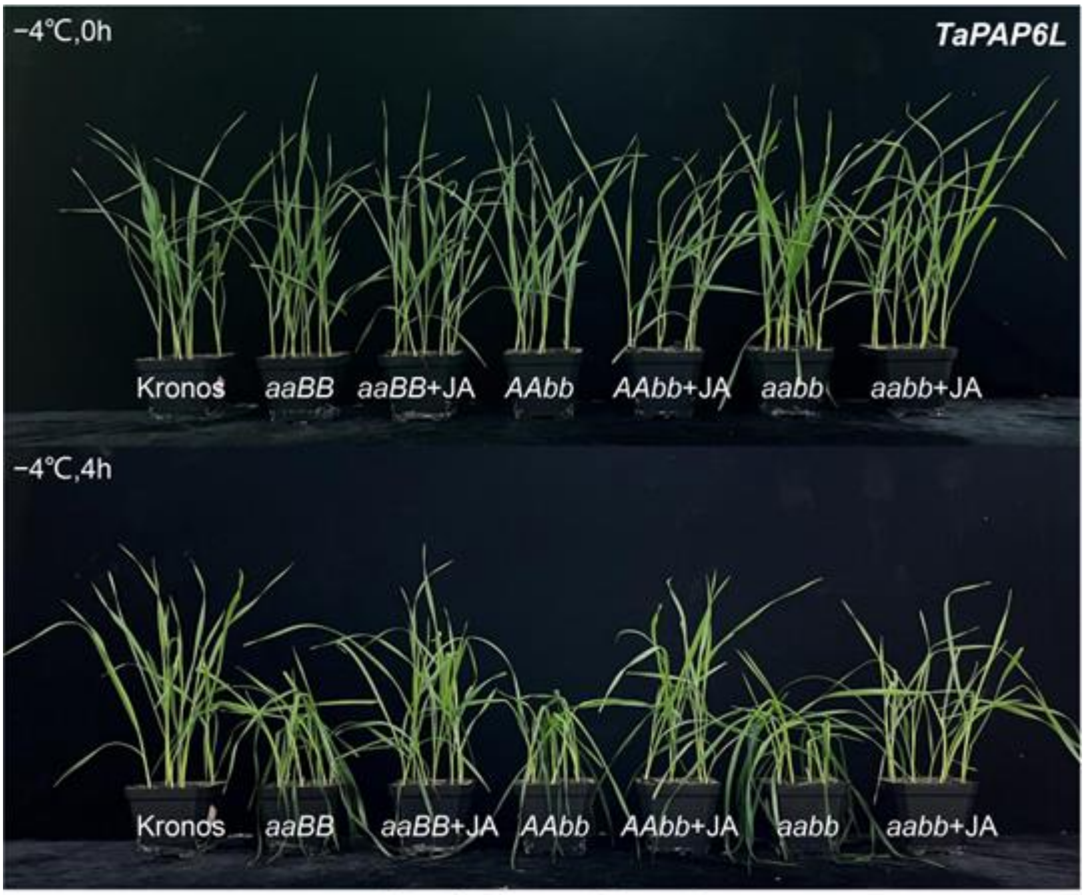

Supplementary Figure 3

Supplemental Figure 4. Exogenous JA (100 μM) significantly enhanced wheat cold tolerance in *TaPAP6L-2B-OE* lines after cold stress.

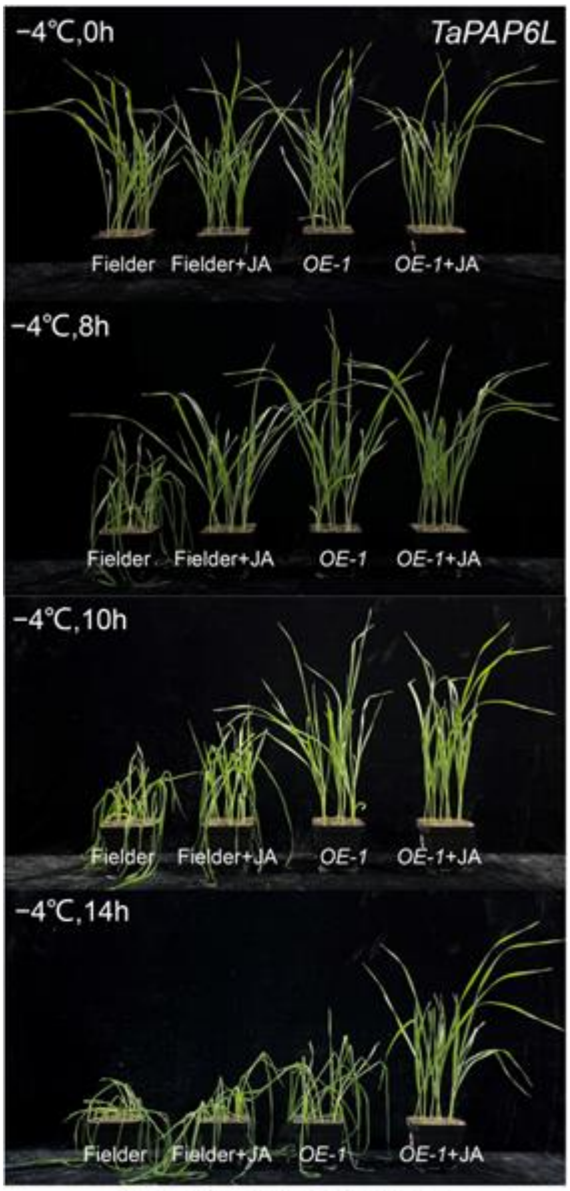

Supplementary Figure 4

Supplemental Table 1. The expressions of 28 annotation genes in a 9-Mb (532-541 Mb) interval between cold-tolerant pools (CTPs) and cold-sensitive pools (CSPs) based on transcriptome analysis.

Supplemental Table 1. The expression of 28 annotation genes in a 9-Mb (532-541 Mb) interval between cold-tolerant pools (CTPs) and cold-sensitive pools (CSPs) based on transcriptome analysis.

Gene No.	Gene_ID	Position	Combination contrast			
			S_{Cold}/S_{CK} (24h)	S_{Cold}/S_{CK} (48h)	S_{Cold}/T_{cold} (24h)	S_{Cold}/T_{cold} (48h)
	AX-109502088	530.3199				
	AX-109569364	532.2137				
	AX-110494163	532.232				
	AX-108958046	532.2649				
1	TraesCS1A02G345900	532.778	-	-	+	-
2	TraesCS1A02G346600	533.140	-	-	+	-
3	TraesCS1A02G347700	533.448	+	+	-	-
4	TraesCS1A02G348500	533.733	+	+	-	-
5	TraesCS1A02G348700	534.044	+	+	-	-
6	TraesCS1A02G350100	535.028	-	-	+	-
7	TraesCS1A02G350200	535.062	+	+	-	-
8	TraesCS1A02G350500	535.315	+	+	+	+
9	TraesCS1A02G350600	535.324	-	-	+	-
10	TraesCS1A02G350900	535.445	-	-	+	-
	AX-94786525	536.0259				
	AX-94843313	536.0259				
11	TraesCS1A02G351900	536.354	+	+	-	-
12	TraesCS1A02G352700	536.661	+	+	-	-
13	TraesCS1A02G354400	537.354	-	-	+	-
14	TraesCS1A02G354600	537.538	+	-	-	-
15	TraesCS1A02G354700	537.543	+	+	-	-
16	TraesCS1A02G355100	537.828	+	+	-	-
17	TraesCS1A02G355200	537.961	+	-	-	-
18	TraesCS1A02G355500	537.974	+	+	-	-
19	TraesCS1A02G356900	539.760	+	+	-	-
20	TraesCS1A02G357300	539.965	-	+	-	-
21	TraesCS1A02G357400	539.974	-	-	+	+
22	TraesCS1A02G357500	540.048	-	-	+	-
23	TraesCS1A02G357900	540.144	-	-	+	+
24	TraesCS1A02G358000	540.152	-	-	-	+
25	TraesCS1A02G358600	540.337	+	-	-	-
26	TraesCS1A02G358800	540.403	-	-	+	+
27	TraesCS1A02G360300	541.466	+	+	+	+
28	TraesCS1A02G360400	541.628	+	+	+	+
	AX-110923532	541.9993				
	AX-94667209	554.5126				

Supplemental Table 2. Hi-Tom sequencing results of *TaSnRK1α*-edited

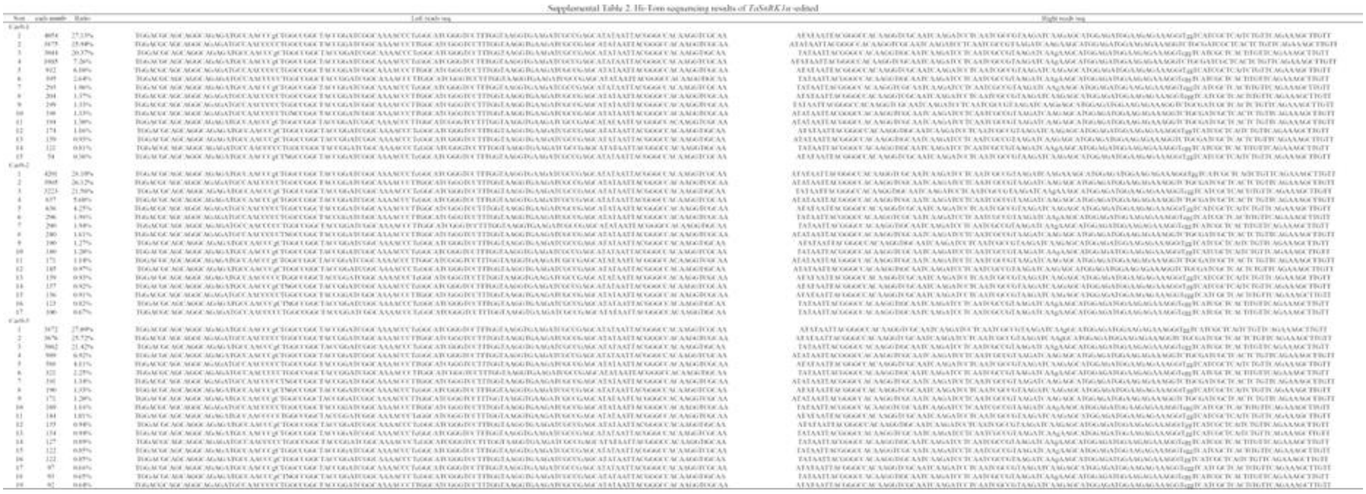
Supplemental Table 2. Hi-Tom sequencing results of [illegible]

Supplemental Table 3. Differentially expressed genes (DEGs) involved in Jasmonic acid-related pathway between *TaPAP6L-2B-OE* plants and Fielder based on transcriptome sequencing.

Supplemental Table 3. Differentially expressed genes (DEGs) involved in Jasmonic acid-related pathway between *TaPAP6L* -overexpressed plants and Fielder based on transcriptome sequencing.

Gene_id	log_2FC	KEGG_Annotation	Description
TraesCS1B02G019800	5.584495	K05894	12-oxophytodienoate reductase 1-like [Aegilops tauschii subsp. tauschii]
TraesCS1D02G111900	1.665799	K00232	acyl-coenzyme A oxidase 4, peroxisomal [Aegilops tauschii subsp. tauschii]
TraesCS3D02G077200	1.700971	K10527	peroxisomal fatty acid beta-oxidation multifunctional protein [Aegilops tauschii subsp. tauschii]
TraesCS4A02G061800	4.889646	K01723	allene oxide synthase 2-like [Aegilops tauschii subsp. tauschii]
TraesCS4A02G061900	2.687687	K01723	unnamed protein product [Triticum turgidum subsp. durum]
TraesCS5A02G007900	3.512015	K00454	Lipoxygenase 2.1, chloroplastic [Triticum urartu]
TraesCS5B02G006500	3.313769	K00454	unnamed protein product [Triticum turgidum subsp. durum]
TraesCS5D02G013400	3.4284	K00454	lipoxygenase 2.1, chloroplastic [Aegilops tauschii subsp. tauschii]
TraesCS6A02G392400	1.735641	K07513	unnamed protein product [Triticum turgidum subsp. durum]
TraesCS6B02G153900	1.631692	K10527	unnamed protein product [Triticum turgidum subsp. durum]
TraesCS6B02G353200	2.789349	K05894	unnamed protein product [Triticum turgidum subsp. durum]
TraesCS6D02G010100	2.498187	K00454	lipoxygenase 2.2, chloroplastic-like [Aegilops tauschii subsp. tauschii]
TraesCS6D02G302300	2.276092	K05894	putative 12-oxophytodienoate reductase 11 [Aegilops tauschii subsp. tauschii]
TraesCS7A02G141800	2.42362	K00232	unnamed protein product [Triticum turgidum subsp. durum]
TraesCS7A02G246200	1.744483	K00454	unnamed protein product [Triticum turgidum subsp. durum]
TraesCS7A02G537100	2.78991	K05894	unnamed protein product [Triticum turgidum subsp. durum]
TraesCS7B02G044200	1.770381	K00232	acyl-coenzyme A oxidase 2, peroxisomal-like [Aegilops tauschii subsp. tauschii]
TraesCS7B02G145200	3.063969	K00454	unnamed protein product [Triticum turgidum subsp. durum]
TraesCS7B02G455300	2.935766	K05894	unnamed protein product [Triticum turgidum subsp. durum]
TraesCS7D02G143200	2.419907	K00232	acyl-coenzyme A oxidase 2, peroxisomal-like [Aegilops tauschii subsp. tauschii]
TraesCS7D02G175800	2.548877	K05894	unnamed protein product [Triticum turgidum subsp. durum]
TraesCS7D02G175900	2.074269	K05894	12-oxophytodienoate reductase 1-like isoform X1 [Aegilops tauschii subsp. tauschii]
TraesCS7D02G176000	1.657162	K05894	unnamed protein product [Triticum turgidum subsp. durum]
TraesCS7D02G244800	2.393356	K00454	probable lipoxygenase 8, chloroplastic [Aegilops tauschii subsp. tauschii]
TraesCS7D02G346400	9.055767	K00232	acyl-coenzyme A oxidase 3, peroxisomal-like [Aegilops tauschii subsp. tauschii]
TraesCS7D02G524400	2.220487	K05894	12-oxophytodienoate reductase 1-like [Aegilops tauschii subsp. tauschii]
Triticum_aestivum_newGene_24021	3.554405	K05894	unnamed protein product [Triticum turgidum subsp. durum]
TraesCS5A02G245200	-2.19214	K14506	unnamed protein product [Triticum turgidum subsp. durum]
TraesCS5B02G242700	-2.92723	K14506	unnamed protein product [Triticum turgidum subsp. durum]
TraesCS5D02G251800	-2.92427	K14506	unnamed protein product [Triticum turgidum subsp. durum]

Supplemental Table 4. All of the primers used in this study.

Supplemental Table 4. All of the primers used in this study

Name	Forward primer (5'-3')	Reverse primer (5'-3')
$BSMV_{TraesCS1A02G360300}$	TTAGCTAGCTGATTAATTAATGGTGAGGTGCGTTTGAAAG	CTTCCGTTGCTAGCTGAGCGGCCGCTTGCTCCCTTCTTCCCGAAT
$BSMV_{TraesCS1A02G360400}$	TTAGCTAGCTGATTAATTAAGTCATCCGCTACTACCCCTC	CTTCCGTTGCTAGCTGAGCGGCCGCAGGCGGGTATGAGCTATGTC
$BSMV_{TraesCS1A02G350500}$	TTAGCTAGCTGATTAATTAATCCTCCAGACACTGCACAA	CTTCCGTTGCTAGCTGAGCGGCCGCAAGGTAGCCACTGGTTGTAC
Actin	GTTCCAATCTATGAGGGATACACGC	GAACCTCCACTGAGAACAACATTACC
RT-TaSnRK1α	CCACAAGGTCGCAATCAAGA	CGACGAGCTTCTTCCTCTTG
RT-TaPAP6L	CGTCGTGGTCATCTTGCTG	AGCTTGATTTTCAGCGAGGG
Kronos331	CTGATTGGCTGTGCTTTCTG	GGTCGATTTGTCACTGAGGC
Kronos4218	TAACCGTATTTGTCACTGTGGA	ACAGAGTAACGCAAATACCTCA
Kronos4220	GGTAGAAAGCACAACCTCTCA	GGTGCTGCATAATTTGGGC
TaSnRK1α-overexpressed	GGATCCCCGGGTACCATGGATTACAAGGATGACGACGATAAGGACG CAGCAGGCAGA	TCGAGCTCTCTAGAACTAGTTCAATGGTGATGGTGATGATGAAGGACTCT CAGCTGGGT
TaPAP6L-overexpressed	GATGACGATGACAAGGGATCCGCCATGGCATCGCCGTCG	GAACGAAAGCTCTGAGCTCTCAATGGTGATGGTGATGATGTGAGACGAC GAACACCCTG
TaSnRK1α_edited_1	AATAATGGTCTCAGGCGCGCCGTAAGATCAAGAGCA	GCGCCGTAAGATCAAGAGCAGTTTTAGAGCTAGAAATAGC
TaSnRK1α_edited_2	GCGATCTTCACCTTACCAACGCTTCTTGGTGCC	ATTATTGGTCTCTAAACGCGATCTTCACCTTACCAA
TaSnRK1α_edited_3	GGAGTGAGTACGGTGTGCCGACGATGGACGCAGCA	GAGTTGGATGCTGGATGGAACAAGCTTTCTGAACA
TaSnRK1α_edited_4	GGAGTGAGTACGGTGTGCTGGACGCAGCAGGCAGA	GAGTTGGATGCTGGATGGAACAAGCTTTCTGAACA
TaSnRK1α-BD	TCAGAGGAGACCTGCATATGGACGCAGCAGGCAGAGATG	CCGCTGCAGGTCGACGGATCCTCAAAGGACTCTCAGCTGG
TaSnRK1α-AD	GTACCAGATTACGCTCATATGGACGCAGCAGGCAGAGATG	CAGCTCGAGCTCGATGGATCCTCAAAGGACTCTCAGCT
TaPAP6L-AD	TACCAGATTACGCTCATATGGCCATGGCATCGCCG	AGCTCGAGCTCGATGGATCCTCATGAGACGACGAA
TaDJ-1-AD	GTACCAGATTACGCTCATATGGCGCCGTCCAAGA	AGCTCGAGCTCGATGGATCCTCAGAAGGAGACGGT
nLUC-TaSnRK1α	GACGAGCTCGGTACCATGGACGCAGCAGGC	CGAGATCTGGTCGACAAGGACTCTCAGCTG
cLUC-TaSnRK1α	TCCCGGGGCGGTACCATGGACGCAGCAGGC	GCTCTGCAGGTCGACAAGGACTCTCAGCTG
nLUC-TaPAP6L	GACGAGCTCGGTACCATGGCCATGGCATCGCCG	CGAGATCTGGTCGACTGAGACGACGAACACC
cLUC-TaDJ-1	TCCCGGGGCGGTACCATGGCGCCGTCCAAGAAGGT	GCTCTGCAGGTCGACGAACGAGACGGTGATGCCCA
pET28a-His-TaSnRK1α	GTGCCGCGCGGCAGCCATATGGACGCAGCAGGCAGAGATG	GTGGTGGTGGTGGTGCTCGAGAAGGACTCTCAGCTGGGTT
pGEX6p-GST-TaPAP6L-Flag	GGCCCCTGGGATCCATGGATTACAAGGATGACGACGATAAGGCCAT GGCATCGCCGTCG	CAGTCACGATGCGGCCGCTCACTTATCGTCGTCATCCTTGTAATCTGAGA CGACGAACA
Ubi::GFP-TaSnRK1α	TGGTGTTACTTCTGCAAAGCTTATGGACGCAGCAGGCAGAG	GCCCTTGCTCACCATGGATCCATGGTGATGGTGATGATGAAGGACTCTCA
Ubi::GFP-TaPAP6L	GGTGTTACTTCTGCAAAGCTTATGGATTACAAGGATGACGACGATA AGGCCAAATTCCA	TCGCCCTTGCTCACCATGGATCCGACCGTGTAAACACCAAATGCGATG
Ubi::GFP-TaDJ-1	TGTTGTTTGGTGTTACTTCTGCAAAGCTTATGGCGCCGTCCAAGAAG GTG	CCTCGCCCTTGCTCACCATGGATCCGAAGGAGACGGTGA
nEYFP-TaSnRK1α	GACTCAGATCACTCGAGATGGATTACAAGGATGACGACGATAAGGA CGCAGCAGGCAGA	AGATCAGGTGGATCCTCAATGGTGATGGTGATGATGAAGGACTCTCAGC TGG
cEYFP-TaPAP6L	CCGGACTCAGATCACTCGAGATGGCCAAATTCCAGATTACAC	ACTCTAGATCAGGTGGATCCTCAGACCGTGTAAACACCAAA
$BSMV_{TaDJ-1}$	TTAGCTAGCTGATTAATTAACACGGCGTTCTTTAGCTTGC	CTTCCGTTGCTAGCTGAGCGGCCGCCGTAGTCGGCACAGAGGAAG
35s::GFP-TaSnRK1α-His	CCTCGAGATGGGTACCATGCATCATCACCATCACCATGACGCAGCA GGCAGAGATGCCA	CCTTGCTCACCATACTAGTATGGTGATGGTGATGATGAAGGACTCTCAGC TGGGTTAGG
35s::GFP-TaPAP6L-Flag	CCTCGAGATGGGTACCATGGATTACAAGGATGACGACGATAAGGCC ATGGCATCGCCGT	CCTTGCTCACCATACTAGTCTTATCGTCGTCATCCTTGTAATCTGAGACG ACGAACACC
GST-TaPAP6L-Flag	GGCCCCTGGGATCCATGGATTACAAGGATGACGACGATAAGGCCAT GGCATCGCCGTCG	CAGTCACGATGCGGCCGCTCACTTATCGTCGTCATCCTTGTAATCTGAGA CGACGAACA
GST-TaSnRK1α	GGGCCCCTGGGATCCATGCATCATCACCATCACCATGACGCAGCAG GCAGAGATGCCAA	GTCACGATGCGGCCGCTCAATGGTGATGGTGATGATGAAGGACTCTCAG CTGGGTTAGGA
GST-TaGRIK1	GGGCCCCTGGGATCCATGGCAGACCTCACGGACAT	TCAGTCACGATGCGGCCGCTTATTGTACTGCTTCT
Kronos2368	TCGCTTGATGACGGATGCTC	AAGACTTCTGTTTCGGGGCA
Kronos4490	CCTGCCACGACTCACCTAAG	CGTGCAGAATCGACAGGTGT
$GST-TaPAP6L^{R30S}-Flag$	CCCGCGAGCAGCAAGCGCAGCAACCTATGGC	GCTGCGCTTGCTGCTCGCGGGAGGACTAGGC
$GST-TaPAP6L^{R38S}-Flag$	ACCTATGGCGGGCAAGCAGCAGCAGGAGGAG	TGCTGCTTGCCCGCCATAGGTTCCTGCGCTT
